# Spatiotemporally Heterogeneous Population Dynamics of Gut Bacteria Inferred from Fecal Time Series Data

**DOI:** 10.1128/mBio.01453-17

**Published:** 2018-01-09

**Authors:** Hidetoshi Inamine, Stephen P. Ellner, Peter D. Newell, Yuan Luo, Nicolas Buchon, Angela E. Douglas

**Affiliations:** a Department of Ecology and Evolutionary Biology, Cornell University, Ithaca, New York, USA; b Department of Entomology, Cornell University, Ithaca, New York, USA; c Department of Molecular Biology and Genetics, Cornell University, Ithaca, New York, USA; Palo Alto Health Care System

**Keywords:** *Acetobacter tropicalis*, *Drosophila melanogaster*, gut microbiota, microbial ecology, population dynamics

## Abstract

A priority in gut microbiome research is to develop methods to investigate ecological processes shaping microbial populations in the host from readily accessible data, such as fecal samples. Here, we demonstrate that these processes can be inferred from the proportion of ingested microorganisms that is egested and their egestion time distribution, by using general mathematical models that link within-host processes to statistics from fecal time series. We apply this framework to *Drosophila melanogaster* and its gut bacterium *Acetobacter tropicalis*. Specifically, we investigate changes in their interactions following ingestion of a food bolus containing bacteria in a set of treatments varying the following key parameters: the density of exogenous bacteria ingested by the flies (low/high) and the association status of the host (axenic or monoassociated with *A. tropicalis*). At 5 h post-ingestion, ~35% of the intact bacterial cells have transited through the gut with the food bolus and ~10% are retained in a viable and culturable state, leaving ~55% that have likely been lysed in the gut. Our models imply that lysis and retention occur over a short spatial range within the gut when the bacteria are ingested from a low density, but more broadly in the host gut when ingested from a high density, by both gnotobiotic and axenic hosts. Our study illustrates how time series data complement the analysis of static abundance patterns to infer ecological processes as bacteria traverse the host. Our approach can be extended to investigate how different bacterial species interact within the host to understand the processes shaping microbial community assembly.

## IMPORTANCE

A major challenge to our understanding of the gut microbiome in animals is that it is profoundly difficult to investigate the fate of ingested microbial cells as they travel through the gut. Here, we created mathematical tools to analyze microbial dynamics in the gut from the temporal pattern of their abundance in fecal samples, i.e., without direct observation of the dynamics, and validated them with *Drosophila* fruit flies. Our analyses revealed that over 5 h after ingestion, most bacteria have likely died in the host or have been egested as intact cells, while some living cells have been retained in the host. Bacterial lysis or retention occurred across a larger area of the gut when flies ingest bacteria from high densities than when flies ingest bacteria from low densities. Our mathematical tools can be applied to other systems, including the dynamics of gut microbial populations and communities in humans.

## INTRODUCTION

Many animals harbor a microbial community in their gut (1) that is diverse and variable over time both in one animal host and among different hosts. The microbiota can influence many important phenotypic traits of its animal host, including nutrition, immunity, and behavior (2–6). In turn, the diversity and abundance of microorganisms are influenced by host traits, especially the immune system, interactions among microbes, and the availability of microbial taxa in the external environment (7–11). There is increasing interest in applying ecological concepts to elucidate the processes underlying within-host microbial population and community patterns (e.g., demographic processes, competition, and migration [11–14]). Analysis of the temporal dynamics of populations is particularly valuable, for example, to infer the processes underlying demographic fluctuations and to discriminate between niche and neutral theories of community assembly (15, 16). Generally, past studies have used the microbial composition of fecal samples as a convenient proxy for within-gut processes (e.g., 17–19), with time series data obtained by repeated fecal sampling from individual hosts, although a few systems (notably, the transparent zebrafish larva and *Caenorhabditis elegans*) are amenable to within-gut analysis in real time (20, 21).

The basis for this study was the prediction that the ecological insights that can be gained from analysis of fecal time series data may be constrained by a lack of ecological theory. Our first goal, therefore, was to develop general mathematical models that link within-host ecological processes to statistics that are measurable from microbial abundance in fecal time series data. We then tested the theory by using the amenable gut microbiome system in *Drosophila melanogaster*. This association is facultative both for the host, which can be reared under axenic (germfree) conditions over multiple generations, and for the microbial partners, which are generally readily culturable; standardized associations with one microbial taxon or multiple microbial taxa can be generated by feeding axenic insects on the desired microorganism(s) (22, 23). Previous research has revealed considerable temporal and among-host variations in community composition (24–28), including nonpersistent taxa that transit repeatedly between the fly and food via fecal-oral cycling (29). To facilitate the analysis, our experiments were conducted with a monoassociation, i.e., with a single bacterial partner, and we investigated how the bacterial population dynamics within the host are altered by the density (high/low) of administered bacteria and the microbial status (gnotobiotic/axenic) of the host. We predicted that the axenic fly gut, empty of competitors, may be more readily colonized than that of gnotobiotic flies; and that flies that ingest large numbers of bacteria (high density) are likely to display a stronger immunological response, suppressing bacterial colonization, than flies that ingest fewer bacteria (low density). On the basis of the first experiments of this study, we selected a strain of *Acetobacter tropicalis* isolated from *D. melanogaster* for this analysis. Our experiments reveal that the fate of ingested *A. tropicalis* is not uniform; although some cells transit through the gut with food (as previously described [29]), other cells are lost, possibly lysed in the gut, and others are retained for extended periods. Furthermore, the dynamics of egestion time through the gut suggest that the fate of the cells is dictated by processes occurring in a spatially restricted location within the gut.

## RESULTS

### Dynamics of bacterial populations in *Drosophila.*

**FIG 1:**
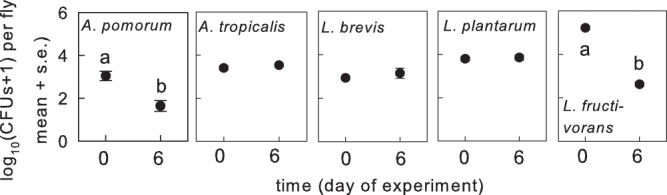
Stability of bacteria in monoassociation with *Drosophila* flies over a 6-day experimental period. *Drosophila* flies were raised from eggs in monoassociation with the bacteria indicated. Density (no. of CFU per fly) is shown at day zero (5-day-old adults) and at day 6 (11-day-old adults) after thrice daily transfers to fresh diet. Different letters indicate significant differences between the two time points.

Our first experiment investigated the stability of bacterial populations in the *Drosophila* gut by using the published procedure of frequent transfers to sterile medium, which depletes the populations of microorganisms with high rates of fecal-oral cycling (29, 30). We reared *Drosophila* flies from birth in monoassociation with five bacterial species of the genera *Acetobacter* and *Lactobacillus* isolated previously from the guts of flies of the same *Drosophila* strain as used in this study (31). At 5 to 6 days after reaching adulthood, the bacterial density in the flies varied significantly between species, from (0.880 ± 0.147) × 10^3^ per fly (*Lactobacillus brevis*) to (175.0 ± 17.1) × 10^3^ per fly (*Lactobacillus fructivorans*) (analysis of variance [ANOVA] on log-transformed data: *F*_4,20_ = 54.6, *P* < 0.001). The flies were then transferred to sterile food thrice daily for 6 days to reduce bacterial cycling between flies and food. The change in bacterial density in the flies varied significantly with the bacterial species (ANOVA interaction term: *F*_4,37_ = 33.47, *P* < 0.001). Analysis by Tukey’s *post hoc* test revealed that the density of three species (*A. tropicalis*, *L. brevis*, and *Lactobacillus plantarum*) did not differ over the 6-day experiment, but that of *Acetobacter pomorum* declined 18-fold and that of *L. fructivorans* declined nearly 200-fold (Fig. 1). These data suggest that the relationship between the bacterial populations in the food and flies varies with the bacterial species. *L. fructivorans* was particularly dependent on oral replenishment, while *A. tropicalis*, *L. plantarum*, and *L. brevis* maintained stable populations in the flies under the experimental conditions used.

**FIG 2:**
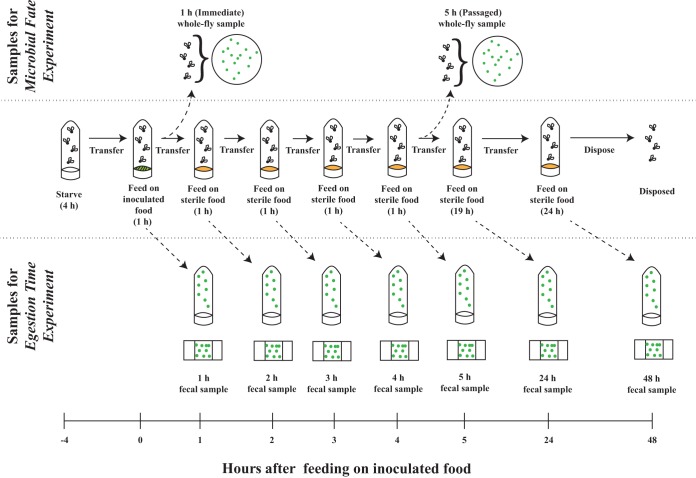
Schematic of the transfer and sampling protocol for the egestion time experiment and the microbial fate experiment. To calculate the egestion time of bacteria and microspheres in the egestion time experiment, each sample of 50 male flies was transferred to a series of fresh vials (solid line). After transfer, fecal samples from the vials were processed and the number of particles was quantified by fluorescence microscope (straight dashed line). To calculate the proportions of ingested bacteria that are egested, retained, and lysed, microbial fate experiment used the same transfer protocol as the egestion time experiment but some samples were sacrificed for CFU counting. The experimental timeline (bottom) shows the number of hours after feeding on food inoculated with GFP-transformed *A. tropicalis*. See Materials and Methods for details.

Our second experiment investigated the short-term dynamics of bacteria that maintain stable populations under thrice daily transfers to a sterile diet. Of the three species with these dynamics (Fig. 1), we focused on *A. tropicalis* because this bacterium is readily amenable to genetic transformation (32). Specifically, we transformed *A. tropicalis* with plasmid pCM62-GFP, which allowed us to track the cells by fluorescence. We confirmed that green fluorescent protein (GFP) expression is stable in *A. tropicalis* for at least 15 days, both in culture and following ingestion by *Drosophila* flies (see Text S1A and Fig. S1 in the supplemental material), and the fluorescing cells are reliably identified by our method (Text S1B and Fig. S2). Our experiments monitored the abundance of the GFP-labeled *A. tropicalis* cells recovered from *Drosophila* feces (Fig. 2). Four treatments were used: axenic or gnotobiotic flies (monoassociated with *A. tropicalis*) were fed on bacteria at high or low density, which enabled us to determine how these factors affect the population dynamics of *A. tropicalis*. To control for variation in the number of bacterial cells ingested and to ensure that the bulk flow of food through the gut was also quantified, fluorescent microspheres that transit through the gut with the food were mixed with the inoculum of bacterial cells.

10.1128/mBio.01453-17.1 TEXT S1  Experimental materials and methods used in this study. Download TEXT S1, PDF file, 0.2 MB. Copyright © 2018 Inamine et al. 2018 Inamine et al. This content is distributed under the terms of the Creative Commons Attribution 4.0 International license.

10.1128/mBio.01453-17.3 FIG S1  Density of *A. tropicalis* colonies (no. of CFU/ml) from fly homogenates plated on mMRS medium with or without tetracycline. Axenic flies were monoassociated with *A. tropicalis*/pCM62-GFP for 15 days to assess the *in vivo* stability of plasmid pCM62. The red dashed line is the 1:1 line. See Text S1A for accompanying text. Download FIG S1, PDF file, 0.1 MB. Copyright © 2018 Inamine et al. 2018 Inamine et al. This content is distributed under the terms of the Creative Commons Attribution 4.0 International license.

10.1128/mBio.01453-17.4 FIG S2  Consistency between manual and automated counts of microspheres and bacteria. Ten microscopy images were chosen at random, and the microspheres and bacteria were counted manually and automatically (with CellProfiler). The red dashed line is the 1:1 line. See Text S1B for accompanying text. Download FIG S2, PDF file, 0.02 MB. Copyright © 2018 Inamine et al. 2018 Inamine et al. This content is distributed under the terms of the Creative Commons Attribution 4.0 International license.

**FIG 3:**
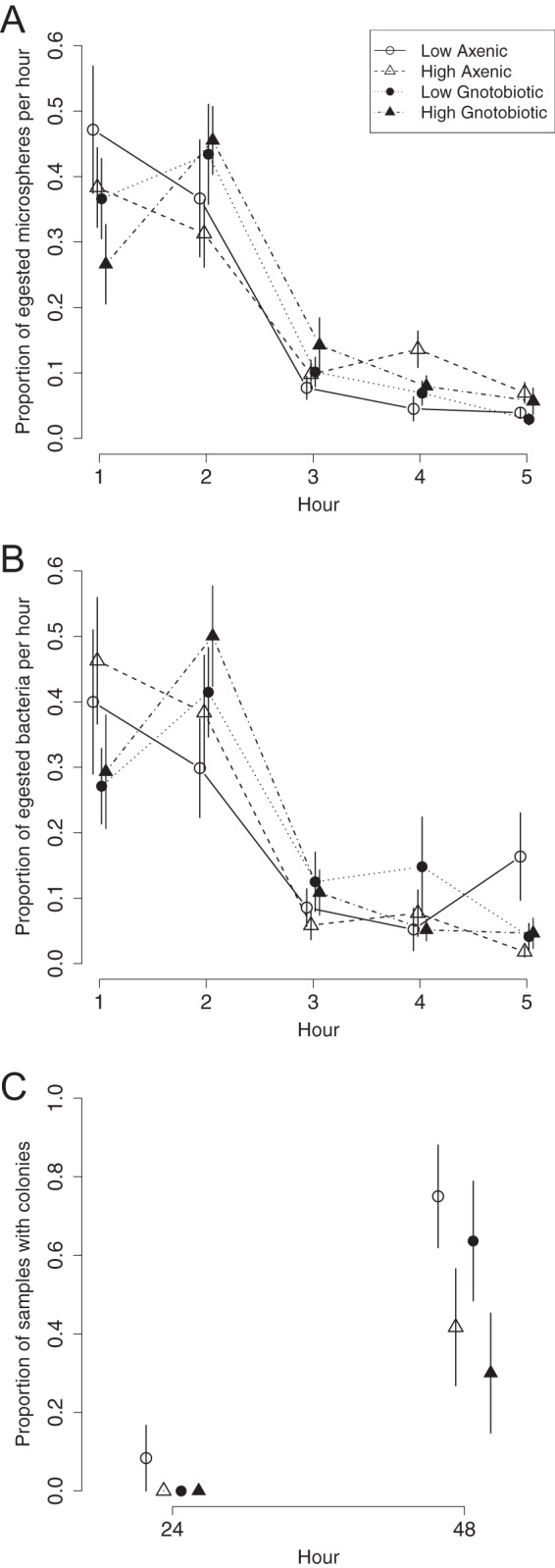
Egestion time dynamics in the egestion time experiment. The proportional mean ± SEM of egested microspheres (A) and bacteria (B) in each sample was calculated by normalizing the number of microspheres (or bacteria) by the total number of microspheres (or bacteria) egested over the initial 5 h, e.g., number of microspheres egested in 1 h divided by the total number of microspheres egested in 5 h. (C) Mean proportion of samples with microbial colonies ± SEM. Samples were scored at 24 and 48 h for the presence/absence of microbial colonies, and the mean proportion of samples was calculated by dividing the number of samples with colonies by the total number of samples with or without colonies. Symbols and colors represent different treatments as follows: open circles, low axenic; open triangles, high axenic; black circles, low gnotobiotic; black triangles, high gnotobiotic.

We scored the abundance of intact GFP-labeled bacteria and fluorescent microspheres in the feces of flies that were transferred hourly to sterile food over 5 h (egestion time experiment, see Fig. 2). Of all the bacterial cells and microspheres egested in the first 24 h, most (a mean of 76%) were egested in the first 2 h and their abundance in the feces tapered to low numbers by 5 h in all treatments (number of cells egested in an hour divided by the total number of cells egested in 5 h; Fig. 3A and B). Very small numbers of GFP-labeled bacteria and microspheres (mean ± standard error of the mean [SEM] = 1% ± 0.3% and 0.4% ± 0.11% of the egested bacteria and microspheres, respectively) were present in the 24-h fecal samples. We inferred that 5 h sufficiently captures the bacteria that are egested with the bulk flow of food. The flies were further cultured to 48 h with a single transfer to sterile food at 24 h (Fig. 2). The condition of bacteria egested from the flies in 48-h samples was different from that of bacteria scored at 1 to 5 h; whereas the bacteria at 1 to 5 h were isolated and easy to count, the bacteria at 48 h were aggregated, making it impossible to score the number of bacterial cells. Using the index of presence/absence of bacteria in each fecal sample, we scored bacterial colonies in 20 to 80% of the fecal samples collected at 48 h, whereas bacteria were detected in only one sample at 24 h (in the low-density treatment administered to axenic flies; Fig. 3C). The proportion of fecal samples collected at 48 h that contained bacteria was significantly larger for flies that had ingested *Acetobacter* bacteria from a low-density inoculum than for flies that had ingested *Acetobacter* bacteria from a high-density inoculum (the number of samples with bacterial colonies divided by the total number of samples is 70 and 36%, respectively; *P* = 0.038 [Fisher exact test]) but did not differ significantly between the axenic and gnotobiotic flies (58 versus 47%; *P* = 0.56 [Fisher exact test]).

Taken together, our results indicate that some *A. tropicalis* cells pass through the host intact with the bulk flow of food but a proportion of ingested cells is retained, giving rise to the bacterial cells that are shed at a later time. These data suggest that population processes occurring in the first few hours after ingestion play a crucial role in the overall dynamics of the *A. tropicalis* populations in the *Drosophila* gut. To guide this analysis, we constructed mathematical models of the population dynamics of microorganisms in the gut.

### Theoretical predictions: ecological inference from the mean and variance of particle egestion times.

To understand how microorganisms interact with the host gut, we developed mathematical models to derive statistics that measure the population dynamics of the microorganisms in the gut. We constructed two classes of models: compartment models and a structural model. Compartment models are differential equation models with specific functions and parameters describing reproduction, death or retention, and movement of microorganisms (such as bacteria) within the gut. The assumptions needed for these models are time-invariant per capita net reproductive rates, migration rates, and unidirectional migration such that a microorganism can travel from compartment *i* to *i +* 1 but not in reverse. This is the simplest set of assumptions needed to build a compartment model. We built a series of compartment models and derived formulas for the mean (μ) and variance (σ^2^) of microbial egestion times as a function of model parameters (Text S2A and Fig. S3 and S4). The structural model is a generalization of the compartment models with qualitative assumptions rather than fully specified process rate functions; e.g., we assumed that the number of ingested microorganisms egested eventually tapers down to 0 and the net reproductive rate of a microorganism decreases the longer it stays in the gut (see Text S2B for technical details). These qualitative assumptions allowed us to test specifically the effect of the decreasing net reproductive rate, irrespective of how it decreases. We used this model to show that our qualitative results from the compartment models hold widely across different models (Text S2B and Fig. S5). Our models do not distinguish between the death and retention of a microorganism in the gut because we assumed in both cases that it disappears from the bulk flow in the compartments tracked by our model.

10.1128/mBio.01453-17.2 TEXT S2  Derivation of theoretical egestion time statistics from models. Download TEXT S2, PDF file, 0.4 MB. Copyright © 2018 Inamine et al. 2018 Inamine et al. This content is distributed under the terms of the Creative Commons Attribution 4.0 International license.

10.1128/mBio.01453-17.5 FIG S3  Compartment diagrams illustrating models with uni- and bidirectional migration among the compartments. Formulas for the mean and variance of egestion time were derived in unidirectional models with one (A) and two (B) compartments. (C) The mean and variance of the egestion time were numerically computed (via simulation) for a model with three compartments and bidirectional migration among the compartments. See Text S2A for accompanying text. Download FIG S3, PDF file, 0.04 MB. Copyright © 2018 Inamine et al. 2018 Inamine et al. This content is distributed under the terms of the Creative Commons Attribution 4.0 International license.

10.1128/mBio.01453-17.6 FIG S4  Cumulative distributions of bacterial egestion times and their statistics under simulations with varying parameters. The simulation is composed of the bidirectional migration model with three compartments (Fig. S3C) under two conditions: varying *r* while *m* = 0.25 (left column) and varying *m* while *r* = −0.25 (right column). For each distribution (top row), the mean (middle row) and variance (bottom row) of the egestion time were calculated. See Text S2A for accompanying text. Download FIG S4, PDF file, 0.1 MB. Copyright © 2018 Inamine et al. 2018 Inamine et al. This content is distributed under the terms of the Creative Commons Attribution 4.0 International license.

10.1128/mBio.01453-17.7 FIG S5  Examples of *f* and *g* functions in the structural model. Many other parameter values, as well as vastly different functional forms, satisfy the assumptions of Theorem 2. See Text S2B for accompanying text. Download FIG S5, PDF file, 0.05 MB. Copyright © 2018 Inamine et al. 2018 Inamine et al. This content is distributed under the terms of the Creative Commons Attribution 4.0 International license.

**FIG 4:**
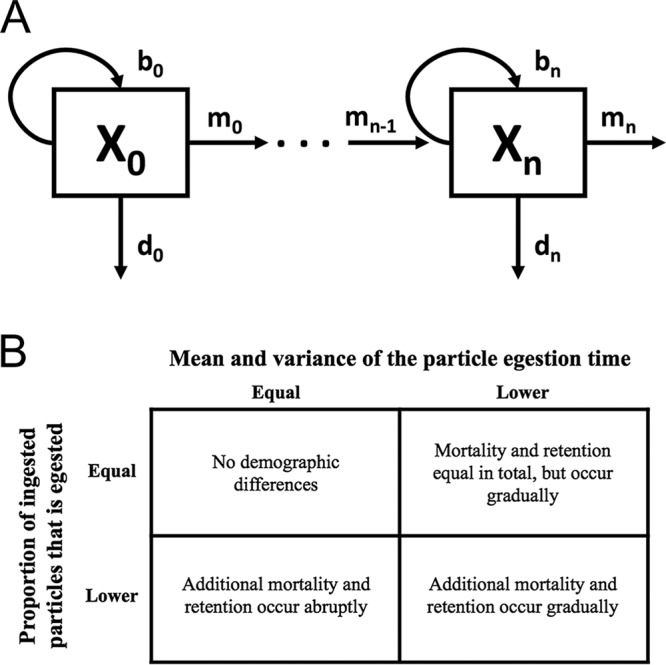
(A) Compartment diagram with arbitrary number of compartments. Each compartment corresponds to a population of bacteria in a gut region (e.g., foregut), and *X*_*i*_ is the microbial population size in the *i*^th^ compartment of the gut. The parameter *r*_*i*_ (= *b*_*i*_ − *d*_*i*_) is the per capita net reproduction rate of the bacteria in the *i*^th^ compartment, and *m*_*i*_ is the per capita rate of migration from the *i*^th^ compartment to the *i* + 1^th^ compartment. (B) Inference scheme based on the compartment model with an arbitrary number of compartments. On the basis of this model, we interpret comparisons within a treatment (bacteria versus microspheres) or between treatments (bacteria versus bacteria). Four demographic patterns can be inferred on the basis of the proportion of ingested particles that is egested and the statistics of the egestion time. A smaller proportion egested and/or a smaller mean and variance of egestion time are expected consequences from different patterns of death or retention in the fly gut.

For a compartment model with *n* + 1 compartments (e.g., foregut, midgut, and hindgut; Fig. 4A), we derived

$$\mu =\sum {}_{i=0}^{n}1/(m_{i}-r_{i})$$

and

$$\sigma^{2}=\sum {}_{i=0}^{n}1/(m_{i}-r_{i}{)}^{2}$$

where *m*_*i*_ and *r*_*i*_ are the per capita emigration rate and net reproduction rate (birth rate *b* minus death or retention rate *d*) in the *i*^th^ compartment, respectively. In our study, we compared the egestion time statistics of a bacterium and ingested microspheres of similar diameters (see “Egestion time statistics for microspheres and bacteria in egestion time experiment” below). For microspheres, which have *r*_*i*_ = 0,

$$\mu =\sum {}_{i=0}^{n}1/m_{i}$$

and

$$\sigma^{2}=\sum {}_{i=0}^{n}1/m_{i}^{2}$$

These formulas show how demographic processes affect μ and σ^2^. Specifically, an increase in the net reproduction rate (i.e., more birth than death or retention) would increase μ and σ^2^, whereas a decrease in the net reproduction rate (i.e., more death or retention than birth) would decrease both. The model also predicts that a decrease in the rate of emigration from compartments would increase μ and σ^2^. Our structural model shows that these results hold for a large class of models and parameters (Text S2B and Fig. S5). Under the additional simplifying assumption that all *n* compartments are identical, the formulas above imply that μ^2^/σ^2^ = *n*.

The results we obtained from theoretical models are intuitive. The more time an ingested microorganism spends in the host, the greater its probability of disappearing in the host (i.e., death or retention) instead of being egested in its feces. When this probability in the host is higher, a microorganism that transits rapidly through the host gut to the feces is more likely to be observed. We therefore expect to see a smaller μ with a higher disappearance rate. Furthermore, an increase in early egestion and a decrease in later egestion should lead to a narrower distribution of bacterial egestion times. We therefore expect to see a lower σ^2^ value with a smaller proportion of ingested bacteria that is egested.

While an increase in death or retention decreases the egestion time statistics, the magnitude of reduction of egestion time statistics depends on how abruptly (over space but also over time because of peristalsis) additional death or retention occurs over the host gut (Text S2C and Table S1). For example, suppose that bacterial death and retention in the host gut reduce the proportion of ingested bacteria that is egested. If death and retention occur gradually (a small effect across many gut compartments), then we observe a large and empirically distinguishable reduction of egestion time statistics (compare the microsphere and C populations in Table S1). However, if death and retention occur abruptly (a large effect in a small number of compartments), then we observe a minor reduction of egestion time statistics that may be difficult to observe empirically (compare the microsphere and B populations in Table S1). Therefore, the proportion of ingested bacteria that is egested and the egestion time statistics can be used together to make inferences about within-host processes without sampling the populations within the host (Fig. 4B).

10.1128/mBio.01453-17.9 TABLE S1  Hypothetical microsphere and four bacterial populations used to illustrate the inference scheme. The set of *r*_*i*_ parameters, the resulting proportion of particles that is egested (%), and the mean (μ) and variance (σ^2^) of the egestion time are shown. For all five populations, *m*_0_ = 5 and *m*_1_ = *m*_2_ = *m*_3_ = *m*_4_ = 1. See Text S2C for accompanying text. Download TABLE S1, PDF file, 0.1 MB. Copyright © 2018 Inamine et al. 2018 Inamine et al. This content is distributed under the terms of the Creative Commons Attribution 4.0 International license.

The intuition behind this inference scheme is as follows. Suppose that ingested microorganisms are subject to very rapid lysis within a very small area of the gut (lysis area) but are unaffected in other areas of the gut (neutral area). The microorganisms then spend most of their time in the neutral area, so the egestion time is approximately equal to the time it takes to pass through the neutral area, regardless of how many die in the lysis area. Egestion time statistics (mean and variance) are therefore nearly independent of the amount of death, even if the fraction that survive is very different. On the other hand, suppose that lysis and/or retention occur broadly throughout the gut. The slower a microorganism passes through a gut region, the less likely it is to escape that region alive. Thus, microorganisms moving faster (e.g., because of bulk flow) will be more likely to emerge in feces. The egestion time distribution is thus biased toward short egestion times (relative to what the distribution would be in the absence of death or retention), with a lower mean and variance of egestion time.

Within treatment, we compare the proportion of ingested bacteria that is egested to the proportion of ingested microspheres that is egested, and egestion time statistics of bacteria (μ_b_ and σ_b_^2^) to microspheres (μ_0_ and σ_0_^2^), to differentiate the bacterial population dynamics from the microsphere dynamics. Between treatments, we compare the proportion of ingested bacteria that is egested and normalized statistics for bacteria (relative to microspheres; μ_norm_ = μ_b_/μ_0_ and σ_norm_^2^ = σ_b_^2^/σ_0_^2^) from one treatment to another to assess the effects of prior interaction of the host with the bacterium (gnotobiotic versus axenic flies) and bacterial density (low versus high) on bacterial population dynamics.

### Proportion of ingested *A. tropicalis* that is egested from the host in egestion time experiment.

**FIG 5:**
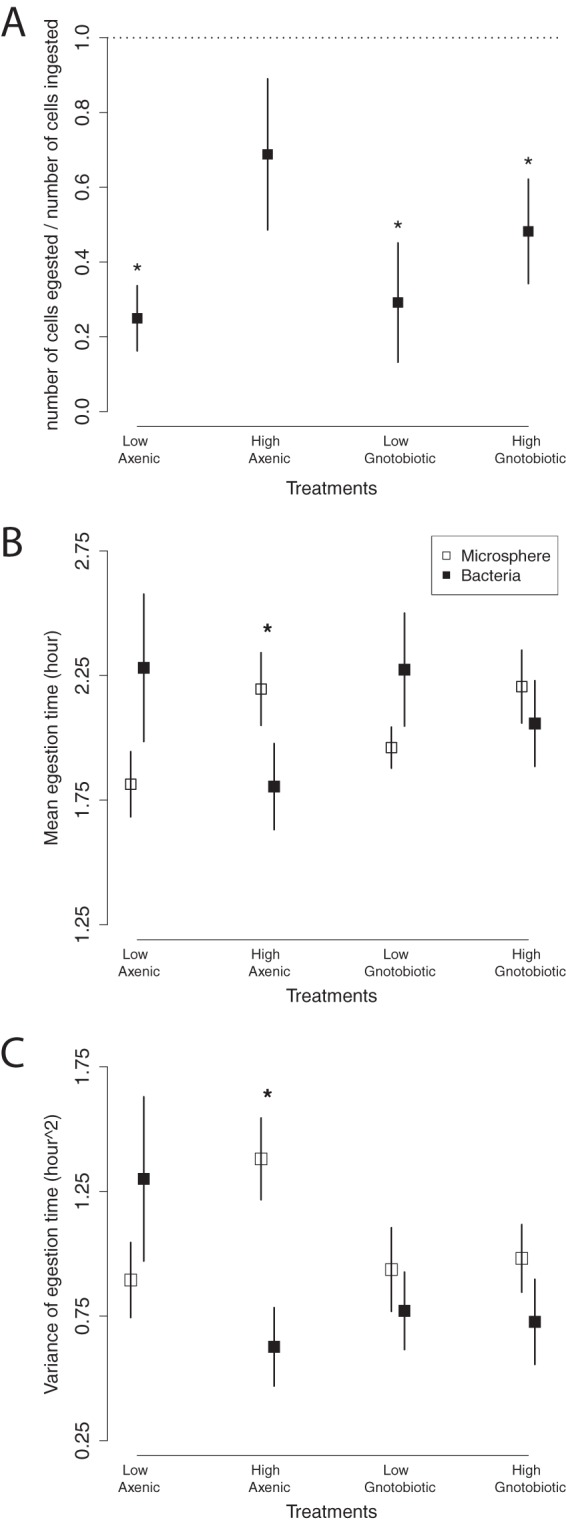
(A) Mean ± SEM of proportion of ingested bacteria that is egested (i.e., the number of bacterial cells egested divided by the number of bacterial cells ingested) in the four different treatments. The dotted line (proportion egested = 1) corresponds to the number of ingested microbial cells predicted from the number of microspheres in the feces. An asterisk indicates that the proportion of ingested bacteria that is egested is significantly <1 (*P* < 0.05). The sample mean ± SEM of mean egestion time (B) and variance of the egestion time (C) of microspheres (□) and bacteria (■) are shown. Statistics were calculated by using the proportional particles egested at each hour (out of the total egested over the initial 5 h; Fig. 3A and B) as the probability distribution of egestion times. See the text for further details. An asterisk indicates significantly different statistics between microspheres and bacteria (*P* < 0.05).

To apply our theoretical predictions, we first calculated the proportion of the *A. tropicalis* cells ingested by the host that was egested (number of egested cells divided by the number of ingested cells; rows in Fig. 4B). Over the 5 h, the total number of egested bacteria was less than the estimated number ingested (Fig. 5A). The proportion of ingested bacteria that is egested was significantly <1 for three treatments: *A. tropicalis* administered at a low density to both axenic flies (LA; mean ± SEM = 0.25 ± 0.12, *P* = 0.003 [*t* test]) and gnotobiotic flies (LG; 0.30 ± 0.18, *P* = 0.02) and at a high density to gnotobiotic flies (HG; 0.45 ± 0.16, *P* = 0.03), but not for *A. tropicalis* administered at a high density to axenic flies (HA; 0.69 ± 0.3, *P* = 0.35). However, no statistically significant differences among the treatments were evident by type II or III ANOVA (bacterial density, *P* = 0.164; axenic/gnotobiotic treatment, *P* = 0.652; interaction, *P* = 0.474) or by stepwise model selection (33) based on the Akaike information criterion (34) and *F* tests. These results demonstrate that a significant proportion of ingested *A. tropicalis* is not detected in the feces, suggesting that 30 to 75% of the ingested *A. tropicalis* cells are retained or lysed within the host, irrespective of the density of ingested cells and prior colonization of the flies with *A. tropicalis*.

### The fate of ingested bacterial cells not egested by the fly: microbial fate experiment.

Following our finding that many ingested bacterial cells do not pass to the feces with the bulk flow of food through the gut (Fig. 5A), we investigated how many ingested *A. tropicalis* cells are retained in the host intact and viable 5 h post-ingestion. We conducted an additional set of experiments (microbial fate experiment, see Materials and Methods and Fig. 2) by using the low-density treatments (LA and LG) described above. The number of viable cells was quantified as the number of CFU on tetracycline-containing plates by using the *tet* gene on the pCM62-GFP plasmid borne by these bacteria, and compared to the total number of bacteria ingested.

**FIG 6:**
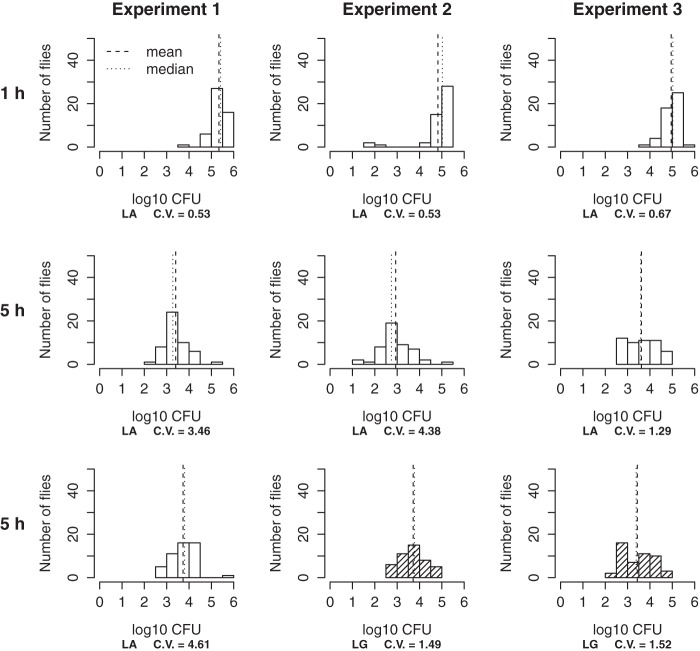
Log_10_ abundance of bacteria retained in the host at 1 h (first row) and 5 h (second and third rows) after the exposure of axenic (white bars) or gnotobiotic (hatched bars) flies to low-density *A. tropicalis* in microbial fate experiments. The three columns correspond to three different replicate experiments performed on different days. Replicate experiment 1 only used axenic treatment, whereas the other replicate experiments used both axenic and gnotobiotic treatments. Each histogram is data from a single vial with 50 flies (LA, low axenic; LG, low gnotobiotic). Vertical dashed and dotted lines are the mean and median, respectively, of log_10_ abundance of bacteria retained in the host body. The coefficient of variation (C.V.) for each vial was calculated by using the untransformed abundance of bacteria retained.

For each of three separate replicate experiments, the number of bacteria per fly was reduced by 1 to 2 orders of magnitude over 5 h in the host body (Fig. 6). Interestingly, the coefficient of variation of the (untransformed) number of bacteria per fly increased over time in each replicate experiment, perhaps reflecting variation in the fate of *A. tropicalis* cells among hosts. Using these results, we estimated the fate of ingested bacteria as follows: ~35% egested, ~10% retained alive in the host, and ~55% lost from the system (inferred to have been lysed, although we cannot exclude the possibility that some retained cells had adopted a viable-but-nonculturable condition) (Table 1; Text S1C to F). Taken together, our data suggest that more than half of the ingested *A. tropicalis* cells are likely lysed during transit through the gut. In the next section, we apply our theoretical models to the egestion time experiment data to investigate the pattern of retention and/or bacterial lysis in the gut.

**TABLE 1:** Fate of *A. tropicalis* ingested by *D. melanogaster* following administration of *A. tropicalis* at low density^a^

Replicate expt	Fly treatment	No. of flies	Proportion of bacteria:		
			Egested	Retained	Lysed
1	Axenic	50	0.11	0.09	0.80
1	Axenic	49	0.10	0.03	0.87
3	Axenic	50	0.26	0.09	0.65
Mean ± SEM			0.18 ± 0.08 (0.12 ± 0.08)	0.08 ± 0.02 (0.07 ± 0.01)	0.74 ± 0.09 (0.81 ± 0.09)
2	Gnotobiotic	45	0.64	0.10	0.25
3	Gnotobiotic	49	0.42	0.16	0.42
Mean ± SEM			0.53 ± 0.11	0.13 ± 0.03	0.34 ± 0.09

a Across three separate replicate experiments, four samples from axenic flies and two samples from gnotobiotic flies were collected and the proportions of ingested bacteria that were egested, retained, and lysed were calculated (e.g., the number of cells egested divided by the number of cells ingested; Text S1C to F). Summary statistics (mean ± SEM) were calculated across the replicate experiments performed over multiple days. The results presented for axenic flies omit one outlier replicate experiment in which no bacteria were recovered from fecal samples. Summary statistics for axenic flies with the outlier included are in parentheses.

### Egestion time statistics for microspheres and bacteria in egestion time experiment.

The mean egestion time of the microspheres and intact GFP-labeled *A. tropicalis* was ca. 2 h. We first used these data to test and validate our models. The mean and variance of the egestion time of the microspheres and bacteria exhibited relationships that are predicted by the models (Text S2C and Fig. S6). We then used the data to infer the second key comparison in the bacterial population dynamics, the pairwise differences in the mean and variance of the egestion time of the microspheres and bacteria in each treatment (columns in Fig. 4B). For gnotobiotic flies, μ_0_ and μ_b_ (the mean egestion time of microspheres and bacteria, respectively) were similar at both bacterial densities (*P* = 0.120 for LG and *P* = 0.293 for HG [paired *t* test]; Fig. 5B). In axenic flies, there was no significant difference at low density (*P* = 0.069 [paired *t* test]) but μ_0_ was significantly higher than μ_b_ at high density (*P* = 0.003 [paired *t* test]; mean difference of 0.39 h with a 95% confidence interval [CI] of 0.164 to 0.619 h). The variance data yielded the same patterns of significance; the variance of the microspheres and bacteria (σ_o_^2^ and σ_b_^2^) did not differ in gnotobiotic flies (*P* = 0.525 and 0.190, respectively [paired *t* test]; Fig. 5C) or in axenic flies at low density (*P* = 0.15 [paired *t* test]), but σ_0_^2^ was significantly larger than σ^2^ at high density (*P* < 0.0001 [paired *t* test]; mean difference of 0.754 with a 95% CI of 0.49 to 1.01).

10.1128/mBio.01453-17.8 FIG S6  Testing predictions based on our theory by using experimental and simulated data. Egestion time statistics were calculated by using data from simulations based on our theory (left column) and from experiments (right column). Both microspheres (□) and bacteria (■) from our experiments were used. Top row: theory predicts a positive correlation between the mean and variance of the egestion time. Bottom row: theory predicts a coefficient of variation (C.V.) of ≤1 (dashed line). See Text S2C for accompanying text. Download FIG S6, PDF file, 0.2 MB. Copyright © 2018 Inamine et al. 2018 Inamine et al. This content is distributed under the terms of the Creative Commons Attribution 4.0 International license.

Combining the data for the proportion of ingested particles that is egested (Fig. 5A) and the mean and variance of the egestion time (Fig. 5B and C), we inferred key processes shaping the population dynamics of *A. tropicalis* in the *Drosophila* gut. Specifically, a proportion of the ingested bacterial cells is retained or lysed abruptly at a small location in the gut for three treatments: *A. tropicalis* administered at high and low densities to gnotobiotic flies and at low density to axenic flies (bottom left cell in Fig. 4B). In contrast, a proportion of the bacteria is retained or lysed gradually across the whole gut when *A. tropicalis* is administered at high density to axenic flies (top right cell in Fig. 4B). These results suggest that microbial population dynamics differ depending on the treatment. This variation could not have been detected from just the total number of bacteria egested.

As noted above (“Theoretical predictions: ecological inference from the mean and variance of particle egestion times”), under the extreme simplifying assumption of identical compartments, the egestion time statistics yield an estimate of the number of independent compartments implied by the data, *n* = μ^2^/σ^2^. Using the mean values (squares) from Fig. 5B and C, we get estimates of *n* = 4.05 ± 0.65 (standard deviation [SD]) from microspheres and *n* = 5.44 ± 1.15 (SD) from bacteria. These values are consistent with the major physiological divisions of foregut; anterior, middle, and posterior regions of midgut; and hindgut.

To investigate further how the population dynamics of *A. tropicalis* vary with treatment, we compared the normalized egestion time statistics (relative to microspheres; e.g., μ_norm_ in HA = μ_b_ in HA/μ_0_ in HA) across the treatments. μ_norm_ is significantly reduced in flies administered *A. tropicalis* at high density relative to low density (*P* < 0.001 for type II and III ANOVAs; slope = −0.32 in simple linear regression, *P* < 0.001), but neither the axenic/gnotobiotic treatment (*P* = 0.826 for type II ANOVA and *P* = 0.873 for type III ANOVA) nor the interaction term (*P* = 0.199 for type II and III ANOVAs) is significant. This pattern is also obtained for σ^2^_norm_ (bacterial density, *P* < 0.005 for type II and III ANOVAs; slope = −0.82 in simple linear regression, *P* < 0.002; axenic/gnotobiotic treatment, *P* = 0.549 for type II and *P* = 0.574 for type III ANOVA; interaction, *P* = 0.434 for type II and III ANOVAs). Taken together, these data suggest that lysis or retention of *A. tropicalis* in the gut is spatially more widespread at high inoculum density, irrespective of whether the flies are gnotobiotic or axenic (Fig. 4B).

## DISCUSSION

The high diversity of microorganisms associated with most animals presents a complex system to decipher, as the interactions between the host and microorganisms are spatiotemporally heterogeneous. To understand the drivers structuring these communities, there is increasing interest in applying ecological theory to microbiomes. Cross-sectional studies based on static abundance or presence data highlight important ecological processes (14, 35), but replicate time series from repeated measurements are becoming especially valuable for the differentiation of ecological processes from stochastic fluctuations within and among hosts (15). Fecal time series are widely collected (18, 19) and contain information on bacterial species interactions (16). However, fecal communities can differ from within-gut communities and lack spatial information (17). The novel mathematical methods developed in this study provide the basis to explain fecal time series as patterns resulting from within-host processes. Specifically, we were able to use the relationship between the spatial processes in the host and the temporal patterns in the feces to infer how spatiotemporal interactions between the *Drosophila* host and its microbiota vary in controlled experiments.

Our results come with two potential caveats. First, we calculated the number of bacteria retained in the host by counting the culturable bacteria. We therefore would have missed viable but nonculturable (VBNC) cells. Although this issue cannot be excluded, its significance is likely minimal because we have not detected VBNC cells of the strain of *A. tropicalis* used in this study under a range of conditions. Second, in our compartment models, we assumed that parameters are constant over time. This may not be fully accurate because the ingested bacterial cells may induce immunological responses in the gut, including a rapid increase in reactive oxygen species and, more slowly, antimicrobial peptide production (36). The magnitude and time scale of these immune responses are not well known, making it difficult to quantify the extent to which our system deviates from the expectations of temporal stability. However, an indication that our theoretical results are robust to the assumption of constant parameters comes from our structural model, which shows that our qualitative results hold for a wide range of parameters and functions, including time-varying parameters (Text S2B and Fig. S5).

Despite substantial research on the gut microbiome in recent years, the population dynamics of gut microorganisms are very poorly resolved. Our theoretical results show that the complexity of the gut habitat can affect the dynamics of microbial populations, with substantial effects of the spatial distribution of host processes on egestion time distributions (Fig. 4B). The implications of these effects are illustrated by our empirical data on *Drosophila*, which indicate that many ingested bacterial cells are apparently lysed over a limited spatial scale. A strong candidate site is the proximal acidic region of the *Drosophila* gut (analogous to the mammalian stomach), which has been demonstrated to suppress the populations of both *Lactobacillus* and *Acetobacter* bacteria (37). However, the lower mean and variance of bacteria (relative to microspheres) at high density (relative to low density) indicate that high numbers of ingested bacteria can lead to a more gradual population reduction as bacteria pass through the gut (top right cell in Fig. 4B). We hypothesize that high bacterial density triggers inducible host immune responses through multiple compartments of the midgut (27, 38, 39), in addition to the constitutive low pH in the acidic region. An induced host immune response at high bacterial density may also explain the smaller proportion of 48-h samples with bacteria present, compared to 48-h samples from low-density treatments.

The high apparent mortality rate of bacteria in the *Drosophila* gut raises the question of the benefits that *A. tropicalis* may receive from associating with this host. In the laboratory, *Acetobacter* density is depressed in the presence of flies relative to that in fly-free vials (25). It is possible that *Drosophila* flies consume and digest *Acetobacter* as part of their diet (29). However, viable bacterial cells are consistently present in feces for hours to days post-ingestion, suggesting that *Acetobacter* may benefit from host-mediated dispersal of viable microbial cells (40; this study). *Drosophila* flies could act as a vector that transfers *A. tropicalis* between ephemeral resources, e.g., rotting fruit or decaying vegetable matter, thereby buffering the bacteria from regional extinction (41). The relationship between *Drosophila* flies and *Acetobacter* bacteria may therefore be antagonistic (host nutritional benefit from bacterial prey) at the level of the individual bacterial cell but mutualistic (host-mediated dispersal of bacteria) at the level of the bacterial population. The relative significance of processes operating at different ecological scales may vary with the ecological circumstances (e.g., composition and availability of food sources) and genotypes of the host and bacteria.

The distribution of the number of ingested bacteria retained among hosts broadens over time (Fig. 6), implying considerable variation in the egestion and lysis of ingested bacteria among hosts. This result parallels the previous finding of considerable variation in the composition of the gut microbiota among hosts (24). Dissecting the causes of variation in the fate of the ingested bacteria and linking this variation to the variation in the composition of the gut microbiota may help us understand how the host-microbiota association changes over time and among hosts.

The model developed here can also be applied to other systems where shedding of ingested bacteria in the feces declines over time. The model, under the extreme simplifying assumption of identical gut compartments, allows researchers to estimate the number of gut compartments from the egestion time statistics (*n* = μ^2^/σ^2^ = 5). However, a more fruitful approach (without the need for this extreme assumption) will be to test the changes in bacterial dynamics across the entire gut. Specifically, our approach identifies the spatial complexity of the interactions between the host gut and ingested bacteria (e.g., lysing bacterial cells across a small area of the gut versus across the entire gut; Fig. 4B). For example, studies of egestion time distributions might provide information about interactions between two bacteria in a host. How would the presence of one bacterial strain affect the egestion time statistics of another? Competition for resources (e.g., space, nutrients), as well as interference competition through toxin production, should lead to a lower mean and variance of egestion time. On the other hand, cooperation and mutualism, both with other microbial taxa and with the host, are predicted to lead to a higher mean and variance of egestion time. We further categorized population dynamics into four different spatiotemporal modes based on the proportion of ingested bacteria that is egested and the mean and variance of the egestion time. This approach highlights aspects of the data set that were previously unstudied and identifies biologically important patterns in fecal time series (e.g., Fig. 3B). Our framework thus allows us to investigate how a microbial population behaves in a multispecies community and, furthermore, the spatial extent of the interaction along the host gut. For example, our approach can be used to answer the question of the extent to which two bacterial species spatially overlap and compete over resources in the host. Building simple microbial communities and investigating them through egestion time provides the basis for an improved mechanistic understanding of the gut microbial ecology in various systems.

## MATERIALS AND METHODS

### Within-host microbial population models.

We constructed models for the dynamics of microbial population size within the host gut (Fig. 4A) where *X*_*i*_ is the microbial population size in the *i*^th^ compartment of the gut (e.g., foregut, midgut, hindgut), *r*_*i*_ is the per capita net reproductive rate of the microorganisms in the *i*^th^ compartment, and *m*_*i*_ is the rate of migration from the *i*^th^ compartment to the *i* + 1^th^ compartment (except in the final compartment, where the microorganisms emigrate to feces). Initially, we assumed that microorganisms flow unidirectionally through an indefinite number of compartments in the host digestive tract. Within each compartment, microorganisms are assumed to have constant per capita birth, death, and emigration rates. In Text S2A, we show the model equations and derive the predicted mean and variance of the microorganism egestion time. These statistics correspond to the mean and variance calculated from time series data for each sample. In Text S2A and B, we further relax the assumptions of unidirectional movement and constant demographic rates to assess whether the same qualitative results hold for a general class of models. We used an *n +* 1 compartment model to categorize inferences based on the proportion of ingested microorganisms that is egested and egestion time statistics (Text S2). It is important to note that our models do not distinguish between the death and retention of a bacteria within a host, since a bacterium disappears in both cases from the compartments tracked by our model. We performed an additional set of experiments to tease apart the proportions of ingested bacteria that are lysed and retained (see Text S1C to F).

### **Culturing of** *Drosophila* **flies and** *A. tropicalis**.***

*Wolbachia*-free *D. melanogaster* Canton-S flies were reared at 25°C on a 12-h–12-h light-dark cycle and a yeast-glucose diet (100 g/liter brewer’s yeast [inactive; MP Biomedicals], 100 g/liter glucose [Sigma], 12 g/liter agar [Apex], and preservatives comprising 0.04% phosphoric acid and 0.42% propionic acid [Sigma]). *A. tropicalis* DmCS006 derived from a single colony was grown overnight on modified MRS (mMRS) medium [12.5 g/liter Bacto peptone, 7.5 g/liter yeast extract, 20 g/liter d-(+)-glucose, 2 g/liter potassium phosphate dibasic trihydrate, 2 g/liter ammonium citrate dibasic, 5 g/liter sodium acetate, 0.1 g/liter magnesium sulfate, 0.05 g/liter manganese sulfate] and concentrated to 10^8^ CFU/ml in sterile phosphate-buffered saline (PBS). Axenic and gnotobiotic flies were prepared as previously described (31). About 30 surface-sterilized eggs were transferred to sterile food per vial. For each gnotobiotic fly vial, a 50-μl suspension of *A. tropicalis* at 10^8^ cells/ml of PBS was transferred directly onto the eggs.

All experiments were conducted with male flies at 5 days post-eclosion. One day before each experiment, axenic and gnotobiotic flies were sexed over sterile chilled aluminum foil and 50 male flies per vial were transferred to freshly prepared sterile food. Female flies were homogenized in sterile PBS, and the suspension was spread onto MRS plates. The presence/absence of *A. tropicalis* was determined by the presence/absence of bacterial colonies on the plate. Only axenic flies from vials with axenic females and gnotobiotic flies from vials with bacterial colonies were used for the experiments described here.

### **GFP-labeled** *A. tropicalis**.***

GFP-labeled, tetracycline-resistant *A. tropicalis* (*A. tropicalis*/pCM62-GFP) cells were created from *A. tropicalis* DmCS006, and the plasmid was confirmed to be stable (see Text S1A). The colonies were streaked onto mMRS plates with 5 μg/ml tetracycline [Sigma-Aldrich] to select for the GFP-expressing strain. A single colony was grown overnight in mMRS without antibiotics, collected by centrifugation, and then resuspended in PBS at the desired density.

### Egestion time experiment.

To control the feeding rate across samples, we adopted a protocol to obtain synchronous feeding on similar volumes of food regardless of the bacterial dosage (42) with minor modifications. Briefly, at the beginning of the light period, the flies were transferred to sterile 50-ml Falcon tubes and starved for 4 h without food or water at 29°C to synchronize and maximize the feeding rate across samples (−4 h to 0 h in Fig. 2). For each sample, 100 μl of a dosing solution consisting of *A. tropicalis* at 10^8^ or 10^9^ CFU/ml (low- and high-density treatments, respectively), microspheres (1.0-μm-diameter blue-green-fluorescent FluoSpheres polystyrene microspheres [F-13080; Life Technologies, Inc.]) at 10^9^/ml, and 2.5% sucrose were laid and dried on solidified sterile *Drosophila* food placed within the Falcon tube cap. *A. tropicalis* cells and the microspheres are similar in size (0.5 to 0.7 by 1.8 to 2.0 μm and 1 μm in diameter, respectively). After starvation, each sample was exposed to the above-described treatment for an hour (0 h to 1 h). Preliminary experiments showed (i) that every fly gut stained blue when exposed to Blue no. 1 dye [Sigma] for an hour of feeding after the starvation condition described above, indicating successful synchronized feeding (results not shown) and (ii) that the number of egested microspheres recovered per fly was uniform across treatments, indicating that *Drosophila* flies fed on similar volumes of food (adjusted *P* > 0.74 for all comparisons [Tukey’s *post hoc* test]). After 1 h, the samples were transferred to new sterile vials with sterile food without the dosing solution. Samples repeatedly went through hourly transfer steps until the end of 5 h for the short-term dynamics experiment. The flies were kept in a 29°C incubator throughout the experiment, except during the transfers. The number of surviving flies was recorded at the end of each 1-h interval.

After transfer, the food cap was removed and the interior of each tube was rinsed with 10 ml of PBS. Tubes were reassembled with sterile caps, vortexed for 1 min, and centrifuged at 5,000 rpm for 10 min at 4°C. The supernatant was carefully discarded, and the pellet was resuspended in 100 μl of PBS. Samples were transferred to sterile Eppendorf tubes and stored in 4°C for up to 6 h until microscopy. For the 24- and 48-h samples, the flies were maintained at 29°C with transfer to fresh, sterile food at 5 and 24 h. Samples were processed at 24 and 48 h exactly as the 1- to 5-h samples were, except that the fecal pellets were scored for the presence/absence of bacteria. Only samples with ≥80% fly survival at the end of 48 h were used in the data analysis. The data displayed are derived from seven independent experiments performed on different days.

### Quantification of egested GFP-labeled *A. tropicalis* and fluorescent microspheres from feces.

Our goal was to quantify the ingested particle (GFP-labeled *A. tropicalis* cells or microspheres) abundance in feces from individual flies over time. We used a Zeiss LSM500 fluorescence confocal microscope and the CellProfiler image analysis software to estimate the number of microspheres or bacteria per sample (43). Specifically, 5 or 7.5 μl of the solution of resuspended fecal matter was mounted on a 24- by 30-mm slide and a field of scope (4,753.7 by 6,040.2 μm) was randomly selected for microscopy. Fluorescent microspheres were identified as blue single fluorescent particles (acquired at a wavelength of 405 nm), and GFP-expressing intact bacteria were identified as green single fluorescent particles (acquired at a wavelength of 488 nm). To maximize precision and reduce spatial variability on a slide, a large area of the slide, consisting of 100 tiles covering a surface of 4,753.7 by 6,040.2 μm, was scanned. The picture tiles were reassembled with Zeiss Zen. The number of particles was then quantified with CellProfiler, supplemented by manual counting for low-quality pictures. The CellProfiler and manual counting methods produced consistent values (Text S1B and Fig. S2). From these measurements, we inferred the number of microspheres and bacteria from the initial sample by (i) scaling up from the area under the field of scope to the total area of the slide, (ii) scaling up to the volume of the aliquot used to prepare the slide (aliquot volume), and (iii) scaling up to the total volume. Particle abundance per fly was therefore calculated as follows:

$$\begin{array}{c} \frac{\#\text{ Particles}}{\text{Fly}}=\frac{\#\text{ Particles}}{\text{Scope}}\times \frac{\text{Scope}}{4753.7\;\mu \text{m}\times 6040.2\;\mu \text{m}}\times \frac{24\text{ mm}\times 30\text{ mm}}{\text{slide}}\times \frac{\text{slide}}{\text{aliquot volume}}\times \frac{\text{total volume}}{\#\text{ Fly}}\times {\left(\frac{1000\;\mu \text{m}}{1\text{ mm}}\right)}^{2}\\ =\frac{\#\text{ Particles}}{\#\text{ Fly}}\times \frac{\text{total volume}}{\text{aliquot volume}}\times \frac{24\times 30\times 1000^{2}}{4753.7\times 6040.2} \end{array}$$

The particle abundances in the inoculum used in the experiments were also quantified under the field of scope to determine the number of microspheres relative to the number of *A. tropicalis* cells in the solution used for each experiment.

### Proportion of ingested bacteria that is egested from feces in egestion time experiment.

Using the number of bacteria recovered from feces over 5 h, we tested if ingested *A. tropicalis* bacteria are egested passively without net reproduction or retention by the host. Specifically, we tested if the number of bacteria egested was equal to the number of bacteria ingested. Flies were exposed to a mixture of known microbial and microsphere densities in the experiments. Under the assumption that the flies ingested both particle types indiscriminately, the number of cells ingested relative to the number of microspheres ingested equals the number of cells in the inoculum relative to the number of microspheres in the inoculum. Similarly, we assumed that both microspheres and bacteria were collected indiscriminately. Then, the number of ingested cells that is egested relative to the number of ingested microspheres that is egested equals the number of cells collected in the feces relative to the number of microspheres collected. We calculated the number of cells or microspheres recovered from feces by summing the number of *A. tropicalis* cells or microspheres in fecal samples over 5 h. Using the counts of microspheres and bacteria in the inoculum and the collected feces, we calculated the proportion of ingested bacteria that is egested from feces as follows (see Text S1C to F for details):

$$\mathrm{Proportion of ingested bacteria that is egested}=\frac{\text{Number of cells egested}}{\text{Number of cells ingested}}=\frac{\left(\text{Number of cells recovered from feces})/(\text{Number of microspheres recovered from feces}\right)}{\left(\text{Number of cells in inoculum})/(\text{Number of microspheres in inoculum}\right)}$$

The proportion of ingested bacteria that is egested is therefore the change in the number of cells relative to the number of microspheres, as both cells and microspheres pass through the fly gut.

The same inoculum was used repeatedly over multiple samples in a single day. Estimates of the proportion from different samples on the same day are therefore not independent, so we averaged the estimated proportion of ingested bacteria that is egested for each treatment within a replicate experiment date. The data are obtained from three independent experiments performed on different days.

We performed Student’s *t* test to assess if the averaged proportion egested differs from 1, where the number of bacteria egested equals the number of bacteria ingested. We then used ANOVA and model selection to assess whether our experimental treatments had any effect on the variation we observed in the proportion egested across treatments.

### Microbial fate experiment.

To investigate if the ingested bacteria are retained or lysed in the host, we performed a separate set of experiments using low-density axenic and gnotobiotic treatments. The experiment followed the procedure of the egestion time experiment, but the flies were homogenized after feeding and passage (Fig. 2). For each of the three replicate experiments, we had a whole-fly sample that was homogenized immediately after 1 h of feeding (low-density axenic immediate sample) and whole-fly samples that were homogenized after 5 h of hourly passage (low-density axenic and low-density gnotobiotic passaged samples).

We calculated the proportion of ingested bacteria that is egested as in the egestion time experiment. To determine how many ingested bacteria were retained or lysed, we used the number of microspheres recovered to calculate the number of bacteria ingested (Text S1C to F). To quantify the number of bacteria retained in the whole host body, we surface sterilized flies in 70% ethanol and then rinsed them in sterile water. We homogenized the whole fly bodies and used a spiral plater to plate them onto mMRS plates with tetracycline (WASP 2 instrument; Microbiology International), selecting for GFP. Plates were incubated in 25°C, and the colonies (CFU) were counted 2 days after plating. Flies were homogenized individually to quantify interhost variation over time. Homogenate microbial abundances were summed within a sample to calculate the mean *A. tropicalis* abundance per fly.

Microbial abundance values from the fecal samples examined under a microscope (number of cells per fly) and from the whole-body homogenates on medium (number of CFU per fly) differ in units used and in the methods of quantification. To compare the two measurements, we calculated the conversion factor between the two units (Text S1E). We then calculated the proportions of ingested bacteria that are retained and lysed.

### Statistical analysis of egestion time from egestion time experiment.

Our theory showed that the mean and variance of the particle egestion time can be used to infer within-host population dynamics (Fig. 4B; Text S2C and Table S1). We therefore calculated these statistics by using the proportion of egested particles from the initial 5 h for each sample, e.g., the number of microspheres egested in 1 h divided by the total number of microspheres egested in 5 h, and tested if the treatments led to different responses in the host. First, we compared the mean and variance of the microbial egestion time to the microsphere statistics within each treatment. We performed paired *t* tests comparing microbial and microsphere egestion time statistics to understand the treatment effects on *A. tropicalis* with microspheres as controls. Second, we divided the mean and variance of the microbial egestion time by the mean and variance of the microsphere egestion time, respectively, to normalize the statistics across treatments. We performed forward model selection on linear models, as well as ANOVA (types II and III, to account for unbalanced sample sizes across treatments), to compare the normalized microbial statistics across treatments. In forward model selection, we used either the bacterial density or fly treatment as the starting covariate. We used the ANOVA function in R (44) to assess whether an additional covariate significantly improves the model.
