# Supplementary material for: Spatiotemporally Heterogeneous Population Dynamics of Gut Bacteria Inferred from Fecal Time Series Data
Source: mBio. 2018 Jan 9;9(1):e01453-17. doi: 10.1128/mBio.01453-17 (PMC5760738; doi:10.1128/mBio.01453-17)
Supplement: TEXT S1 [file mbo001183662s1.pdf]

Text S1: Experimental Materials and Methods.

Text S1A. Cloning and assessing the stability of pCM62-GFP–*Acetobacter tropicalis*

Genes were amplified by PCR and cloned using enzymes from New England Biolabs as per the manufacturer's recommendations. All products were amplified using Phusion polymerase, cut with restriction enzymes as indicated below, and ligated with T4 DNA ligase to the plasmid pCM62 (1) (prepared by cutting with the same enzymes as product to be cloned) such that the Plac promoter would drive expression of the cloned gene. GFP gene was amplified from pMQ80 template (2) with forward [5'-GCTTGCATGCCTGCAGACTAGTC-3'] and reverse [5'-TAAAAAGCTTCAAGCCGTCAATTGTCTGATTCGTTACC-3'] primers, with an annealing temperature of 59°C, extension time of 1 min, and product cloned with PstI and HindIII. Ligated products were introduced into *Acetobacter tropicalis* by conjugation as described (3). Briefly, bacteria were cultured overnight in potato medium and cells from 0.5 ml of culture harvested by centrifugation. Cells of donor and recipient were washed separately in sterile growth medium twice, re-suspended in a final volume of 50 µl potato medium, then mixed together and transferred to a fresh potato medium plate. After incubation at 30 °C for 16 h, cells were harvested and plated onto YPG medium (0.5% yeast extract, 0.5% peptone, 1% glycerol, 1.5% agar) containing 0.2% acetic acid and 20 mg/l chlortetracycline. Colonies that appeared after 48 h of incubation were sub-cultured on potato medium supplemented with 20 mg/l chlortetracycline.

We assessed the stability of pCM62-GFP in *A. tropicalis* by 2 different methods. First, we assessed the *in vitro* stability of GFP expression in the absence of antibiotic selection. If the plasmid were unstable, the bacteria would lose GFP expression as the tetracycline resistance is unnecessary in the absence of antibiotic selection. *A. tropicalis* with pCM62-GFP were serially passaged in MRS without tetracycline. Microbial cultures underwent daily 1:1000 dilution over 5 days. At the end of 5 days, we manually counted the number of *A. tropicalis* under fluorescent microscope, with and without fluorescence. Across samples, 90.44 ± 0.07% of A. tropicalis cells (n=5 independent serial passages) expressed fluorescence, indicating that the bacteria retain pCM62-GFP in vitro.

Second, we assessed the *in vivo* stability of *A. tropicalis* in retaining pCM62 plasmid. Axenic flies were mono-associated with pCM62-GFP-*A. tropicalis* for 15 days, and fly homogenates were plated on MRS media with and without tetracycline. Number of colonies were compared between the two plates to assess the retention of pCM62-GFP. If the bacteria lose the plasmid, then they lose the tetracycline resistance. We therefore expect to see more bacteria on the plates without tetracycline if the plasmid were unstable. We obtained equal number of colonies on plates with and without tetracycline (Fig. S1), indicating that the bacteria retain pCM62-GFP *in vivo*.

Taken together, these results indicate that the bacteria retain the plasmid even in association with the *Drosophila* host, and GFP is stably present even in the absence of antibiotic selection.

Text S1B. Assessing the quality of the automated counting method

The number of particles (microsphere and bacteria) recovered from feces and in inoculum were quantified using the open-source image analysis software CellProfiler (4) and supplemented by manual counting. CellProfiler allows automated counting by discriminating particles based on size, shape, and color. Parameters for particle detections were determined by comparing automated and manual counts over several images. Parameters were chosen such that the particles were identified appropriately and matched visual inspection. To determine the quality of the automated counts, we randomly chose 10 additional images and quantified the particle abundance by both methods. We saw consistency between the two methods, for both microspheres and GFP-labeled *A. tropicalis* (Fig S2).

Text S1C. Estimating proportions of ingested *A. tropicalis* that are retained by, egested out of, and lost in the fly

Ingested bacteria only have three mutually exclusive fates: 1. Intact bacteria are egested out; 2. Intact bacteria are retained in the host over the experiment; or 3. Bacteria are lost due to lysis. To clarify our calculations, we will walk through the procedure using data values from one of the three replicate experiments in *Microbial Fate Experiment* (first row of Axenic fly treatment, Table 1). We calculate proportion of ingested bacteria that is egested in both *Egestion Time Experiment* and *Microbial Fate Experiment*.

1. The first step is to quantify the number of cells ingested by the fly relative to the number of ingested microspheres. Assuming that flies ingested cells and microspheres indiscriminately, this ratio equals the ratio of cells to microspheres in the inoculum, which was measured in each replicate experiment (0.289 cells/microsphere in this example).

Equation S1.1

$$\frac{\text{Number of cells ingested}}{\text{Number of microspheres ingested}} = \frac{\text{Number of cells in inoculum}}{\text{Number of microspheres in inoculum}} = 0.289\,\frac{\text{cells ingested}}{\text{microsphere ingested}}$$

2. The second step is to quantify the number of ingested cells egested by the fly over 5 h relative to the number of ingested microspheres egested. Assuming that ingested cells and microspheres were recovered indiscriminately from the feces, this ratio equals the number of cells recovered from fly feces relative to the number of microspheres recovered over 5 h.

Equation S1.2

$$\frac{\text{Number of cells egested}}{\text{Number of microspheres egested}} = \frac{\text{Number of cells recovered from feces}}{\text{Number of microspheres recovered from feces}} = \frac{3129.421\ \text{cells / fly}}{95647.944\ \text{microspheres / fly}}$$

$$= 0.0327\,\frac{\text{cells}}{\text{microsphere}}$$

3. The third step is to calculate the proportion of ingested bacteria that is egested. Because microspheres were scarce in our 5-24 h and 24-48 h samples, we assume that the total number of microspheres egested over 5 h equals the total number of microspheres ingested.

Equation S1.3

$$\text{Proportion of ingested bacteria that is egested} = \frac{\text{Number of cells egested}}{\text{Number of cells ingested}}$$

$$= \frac{(\text{Number of cells egested}) / (\text{Number of microspheres ingested})}{(\text{Number of cells ingested}) / (\text{Number of microspheres ingested})}$$

$$= \frac{(\text{Number of cells egested}) / (\text{Number of microspheres egested})}{(\text{Number of cells ingested}) / (\text{Number of microspheres ingested})}$$

$$= \frac{0.0327 \text{ cells / microsphere}}{0.289 \text{ cells / microsphere}} = 0.113$$

4. To calculate proportion of ingested bacteria that is retained in the fly in *Microbial Fate Experiment*, we compare the numbers of bacteria ingested and retained. The number of bacteria ingested is calculated from the number of microspheres ingested, using Equation S1.1. We assume that the number of microspheres ingested equals the number egested over 5 h, because microspheres were scarce in our 5-24 h and 24-48 h samples. Microspheres in feces were counted under a microscope. However, microscopy may only account for a fraction of egested microspheres, as some egested particles are lost under our protocols before microscopy (*e.g.* in the process of washing the vials and pelleting the feces by centrifuge). We therefore need to consider the proportion of particles recovered from feces. In Text S1D below, we derive this proportion for each replicate experiment. Using the proportion of particles recovered (0.034 for this example), we calculate the number of microspheres egested:

Equation S1.4

$$\frac{\text{Number of microspheres ingested}}{\text{fly}} = \frac{\text{Number of microspheres egested}}{\text{fly}}$$

$$= \frac{\text{Number of ingested microspheres recovered from feces / fly}}{\text{Proportion of particles recovered}}$$

$$= \frac{95647.944 \text{ microspheres / fly}}{0.034} = 2813175 \frac{\text{microspheres ingested}}{\text{fly}}$$

Equation S1.1 then gives us the number of bacteria ingested (813007.5 cell/fly in this example).

5. Next we estimate the number of cells retained intact in the host. At the end of 5 h, we homogenized the *Passaged* flies and used a spiral plater to estimate the number of *A. tropicalis* CFU per fly. In Text S1E below, we derive the conversion factor from number of CFU's (spiral plater) to the number of cells scored by fluorescence microscopy (1 CFU/ml = 2.83 bacterial cells/ml). Using this conversion factor,

Equation S1.5

$$\frac{\text{Number of cells retained}}{\text{fly}} = \frac{\text{Number of CFU retained}}{\text{fly}} \times \text{Conversion factor}$$

$$= 25011.8\frac{\text{CFU}}{\text{fly}} \times 2.83\frac{\text{cells}}{\text{CFU}} = 70783.39\frac{\text{cells retained}}{\text{fly}}$$

Then using the numbers of cells retained (Equation S1.5) and ingested, we have

Equation S1.6

$$\text{Proportion of ingested bacteria that is retained} = \frac{\text{Number of cells retained}}{\text{Number of cells ingested}} = \frac{70783.39 \text{ cells/fly}}{813007.5 \text{ cells/fly}} = 0.087$$

6. Lastly, we calculate the proportion of ingested *A. tropicalis* that is lysed by the end of the experiment.

Equation S1.7

$$\text{Proportion of ingested bacteria that is lysed}$$
$$= 1 - (\text{Proportion of bacteria that is egested}) - (\text{Proportion of bacteria that is retained})$$
$$= 1 - 0.113 - 0.087 = 0.8$$

We performed the same calculations for all samples and the results are shown in Table 1. Across both Axenic and Gnotobiotic samples, we observed statistically significant proportions of ingested bacteria that are egested, retained, and lysed (*t*-test against null hypothesis that mean = 0. Mean ± SEM = 0.25 ± 0.10, p=0.048; 0.09 ± 0.02, p=0.005; and 0.66 ± 0.11, p=0.002, respectively). Importantly, proportion of ingested bacteria that is egested are similar between *Egestion Time Experiment* and *Microbial Fate Experiment* (LA: mean ± SEM = 0.25 ± 0.12 and 0.18 ± 0.08, respectively. LG: 0.30 ± 0.18 and 0.53 ± 0.11, respectively), implying consistency between the experiments.

In these calculations, we ignored the possibility of bacteria reproduction in the host. If reproduction is present, then actual proportion of bacteria that is lysed would be higher than our calculated estimate. Suppose that there is some reproduction, z, in the host. Then the actual proportion of bacteria that is lysed is

Equation S1.8

$$\text{Proportion of ingested bacteria that is lysed}$$

$$= \frac{(\text{Number of cells ingested}+z) - (\text{Number of cells egested}) - (\text{Number of cells retained})}{(\text{Number of cells ingested}+z)} = \frac{a+z}{b+z}$$

where a = *Number of cells lysed* and b = *Number of cells ingested*, with $a \leq b$. Equation S1.8 is an increasing function of z. Our estimated proportion of ingested bacteria that is lysed, therefore, is a conservative estimate of the actual proportion. Conversely, some egested and retained cells would have been cells produced in the host. The actual proportions of egested and retained bacteria would then be lower than our estimated proportions.

Finally, what happens to our calculated proportions of ingested bacteria that are egested, retained, and lysed if some of the microspheres were retained by the host? For example, suppose that 50% of ingested microspheres were retained in the fly gut, and only 50% of ingested microspheres were egested. Then in Equation S1.3 for the proportion of ingested bacteria that is egested, we would have

Equation S1.9

$$\text{Proportion of ingested bacteria that is egested} = \frac{\text{Number of cells egested}}{\text{Number of cells ingested}}$$

$$= \frac{(\text{Number of cells egested}) \ / \ (\text{Number of microspheres ingested})}{(\text{Number of cells ingested}) \ / \ (\text{Number of microspheres ingested})}$$

$$= \frac{(\text{Number of cells egested}) \ / \ (\boldsymbol{2 \times \textit{Number of microspheres egested}})}{(\text{Number of cells ingested}) \ / \ (\text{Number of microspheres ingested})} = \frac{0.0327 \text{ cells / microsphere}}{2 \times 0.289 \text{ cells / microsphere}}$$

$$= 0.057$$

The value in Table 1 for the proportion of ingested bacteria that is egested would then be too high by a factor of 2.

The proportion of ingested bacteria that is retained was also calculated using the number of microspheres egested to estimate the number of bacteria ingested, in Equation S1.4 and Equation S1.1. If half the microspheres were retained rather than egested, the estimated number of bacteria ingested would be low by a factor of 2. Then instead of Equation S1.6 for the proportion of ingested bacteria that is retained, we would have

Equation S1.10

$$\text{Proportion of ingested bacteria that is retained} = \frac{\text{Number of cells retained}}{\text{Number of cells ingested}} = \frac{70783.39 \text{ cells/fly}}{2 \times 813007.5 \text{ cells/fly}} = 0.044$$

The value in Table 1 for the proportion of ingested bacteria that is retained also would be too high by a factor of 2.

Therefore, if not all ingested microspheres are egested, the actual values of the proportions of ingested bacteria that are egested and retained would be lower than the values in Table 1, and the values of the proportion of ingested bacteria that is lysed would be higher. However, this has no effect on our qualitative conclusion: some of the ingested bacteria are egested and retained intact, while many are lysed in the host.

Text S1D. Calculating the proportion of particles recovered in *Microbial Fate Experiment*

Here we derive the proportion of particles recovered that was used to generate Table 1, as explained in Text S1C. To clarify the calculation, we will walk through the procedure using data values from the first replicate experiment (first row in Axenic fly treatment, Table 1) of the *Microbial Fate Experiment*.

The proportion of particles recovered was estimated from the *Microbial Fate Experiment* data on Low density axenic (LA) flies. We assume that proportion of particles recovered is a characteristic of each replicate experiment (*i.e.* a result of how vials were washed, how feces were centrifuged, *etc.* on each date). The derived proportion of particles recovered for a replicate experiment was therefore applied to both Axenic and Gnotobiotic flies on that date, to calculate the number of microspheres and bacteria ingested from the number of microspheres recovered from feces (Equation S1.4).

The first step is to calculate the number of cells ingested using the CFU counts in the *Immediate (1 h)* sample. In Text S1E below, we derive the conversion factor to calculate the number of cells under fluorescent microscope from the CFU on spiral plater (1 CFU/ml = 2.83 bacteria cells/ml). We use this conversion factor to calculate the number of *A. tropicalis* cells ingested by the fly from the number of CFU ingested.

Equation S1.11

$$\frac{\text{Number of cells ingested}}{\text{fly}}$$

$$= \frac{\text{Number of CFU ingested}}{\text{fly}} \times \text{Conversion factor} = 270808 \frac{\text{CFU}}{\text{fly}} \times 2.83 \frac{\text{cells}}{\text{CFU}} = 766386.6 \frac{\text{cells ingested}}{\text{fly}}$$

We measured the *bacteria cells : microsphere* ratio in the inoculum used in each replicate experiment. Assuming that the flies ingested microsphere and bacteria indiscriminately, we use this ratio to calculate the number of microspheres ingested by a fly from the number of cells ingested.

Equation S1.12

$$\frac{\text{Number of microspheres ingested}}{\text{fly}} = \frac{\text{Number of cells ingested / fly}}{\text{Number of cells in inoculum / Number of microspheres in inoculum}}$$

$$= \frac{766386.6 \text{ cells / fly}}{0.289 \text{ cells / microspheres}} = 2651857 \frac{\text{microspheres ingested}}{\text{fly}}$$

This is the number of microspheres ingested by a fly in Axenic *Immediate* sample. We assume that a fly in Axenic *Passaged* sample also ingested the same number of microspheres. Furthermore, because microspheres were scarce in our 5-

24 h and 24-48 h samples, we assume that all ingested microspheres were egested by 5 h.

We counted the number of microspheres recovered in hourly samples from the *Passaged* flies, and summing these over 5 h gives the total number of microspheres recovered from the feces (*e.g.* 91194.54 microspheres/fly). We calculate the proportion of particles recovered for a replicate experiment using the number of microspheres recovered and egested.

Equation S1.13

$$\text{Proportion of particles recovered} = \frac{\text{Number of microspheres recovered from feces}}{\text{Number of microspheres egested}}$$

$$= \frac{\text{Number of microspheres recovered from feces}}{\text{Number of microspheres ingested}} = \frac{91194.54 \text{ microspheres/fly}}{2651857 \text{ microspheres/fly}} = 0.034$$

Applying the same calculation to all three replicate experiment of the *Microbial Fate Experiment* gave estimated proportions of particles recovered of 0.034, 0.012, and 0.023.

Note that we calculate proportion of particles recovered using numbers of microspheres recovered from feces (relative to the estimated number of microspheres egested). In the experiments, both microspheres and bacteria were homogeneously distributed on the experimental food, and fecal samples were collected indiscriminately. We therefore assume that the proportion of particles recovered is the same for bacteria as it is for microspheres.

As noted above, we also assume that the same proportion of particles recovered applies to all samples within a replicate experiment, including both Axenic and Gnotobiotic flies. To test this assumption and validate the estimates of proportion of particles recovered, we re-calculate the proportion of ingested bacteria that is egested using the proportion of particles recovered, and compare the results to the values in Table 1. The values in Table 1 did not use the proportion of particles recovered (Equation S1.3). Therefore, if this alternative calculation (AC) leads to similar values as Table 1, then we conclude that our estimate for the proportion of particles recovered is sound. To test our assumption that the proportion of particles recovered (calculated using Axenic flies) also applies to Gnotobiotic flies, we re-calculate the proportion of bacteria that is egested using the Gnotobiotic flies. To clarify AC, we will walk through its calculation using data values from the second replicate experiment (first row in Gnotobiotic fly treatment, Table 1).

In AC, we assume that each fly in *Passaged* samples (including Gnotobiotic flies) ingested the same number of bacteria as Axenic *Immediate* flies. The flies in the *Immediate* sample from our example ingested 108888 CFU/fly. We convert the number of CFU ingested to the number of *A. tropicalis* cells ingested by a fly using the conversion factor.

Equation S1.14

$$\frac{\text{Number of cells ingested}}{\text{fly}} = \frac{\text{Number of CFU ingested}}{\text{fly}} \times \text{Conversion factor}$$

$$= 108888 \frac{\text{CFU}}{\text{fly}} \times 2.83 \frac{\text{cells}}{\text{CFU}} = 308153 \frac{\text{cells ingested}}{\text{fly}}$$

Next, we calculate the number of cells egested from the number of cells recovered in feces.

Equation S1.15

$$\frac{\text{Number of cells egested}}{\text{fly}} = \frac{\text{Number of cells recovered from feces / fly}}{\text{Proportion of particles recovered}}$$

$$= \frac{\text{2588 cells / fly}}{0.012} = 215667 \frac{\text{cells egested}}{\text{fly}}$$

The proportion of ingested bacteria that is egested using numbers of cells egested (Equation S1.15) and ingested (Equation S1.14) under AC is

Equation S1.16

$$\text{Proportion of ingested bacteria that is egested} = \frac{\text{Number of cells egested}}{\text{Number of cells ingested}} = \frac{\text{215667 cells / fly}}{\text{308153 cells / fly}} = 0.7$$

Calculating the proportion of ingested bacteria that is egested with (AC) and without (Equation S1.3) the proportion of particles recovered led to similar values (mean ± SEM = 0.45 ± 0.26 and 0.53 ± 0.11, respectively) across Gnotobiotic samples. We conclude that our calculation of the proportion of particles recovered is sound.

Text S1E. Conversion factor between microscopy (cells/ml) and spiral plater (CFU/ml) bacterial counts

Here we derive the conversion factor between microscopy and spiral plater that was used in Text S1C and Text S1D. Quantification of retained *A. tropicalis* involves two different methods to count the number of bacteria: fecal samples under fluorescent microscopy (measured in cells/ml) and fly homogenate samples on spiral plater (measured in CFU/ml). The two approaches have different ranges for measurable microbial densities. We must calculate the conversion factor between the two methods so that measurements by the two methods can both be used. We calculated the conversion factor by the following. We grew a culture of GFP-labeled *A. tropicalis* overnight, and re-suspended the culture in PBS. We serially diluted the culture, such that some dilutions are within the measurable range for the spiral plater, whereas others are within the measurable range for microscopy. For each serial dilution, we regressed microscopy or spiral plater measurements against the dilution factor of the sample to obtain a slope between measurements and dilution. We thus obtained paired slopes (β for spiral plater against dilution factor and α for microscopy against dilution factor) for each sample:

Spiral plater:

$$\left[\text{Number } \frac{\text{CFU}}{\text{ml}}\right] = \beta[\text{dilution factor}]$$

Microscopy:

$$\left[\text{Number } \frac{\text{cells}}{\text{ml}}\right] = \alpha[\text{dilution factor}] = \left(\frac{\alpha}{\beta}\right)\left[\text{Number } \frac{\text{CFU}}{\text{ml}}\right]$$

We repeated this for 4 samples, obtaining 4 values of α and β. We regressed (through the origin) the slope from microscopy against the slope from spiral plater, to get a conversion factor across all samples (equivalent to α/β). The estimated conversion factor between two units is 1 CFU/ml = 2.83 bacteria cells/ml (simple linear regression: Standard error=0.35, adjusted R^2=0.94, p=0.004). Alternatively, we regressed (through the origin) the slope from spiral plater against the slope from microscopy (equivalent to β/α) and then took its inverse to calculate α/β. We obtained 1 CFU/ml = 2.96 bacteria cells/ml. The two methods result in similar conversion factor. Since we convert the number of CFU to the number of cells, we use 1 CFU/ml = 2.83 bacteria cells/ml for the paper.

Text S1F. Summary of calculations for Table 1

Here we summarize compactly how the quantities estimated in Text S1C, Text S1D, and Text S1E were used to get the numbers in Table 1. Terms in the calculations are in one of three typefaces to distinguish whether they were directly observed or indirectly inferred. Each typeface describes the following:

Normal type: Data collected by directly observing samples (*e.g.* feces from Axenic *Passaged* flies, inoculum used in a replicate experiment).

Italic type: Inferred from calculation using data on same fly type only.

Bold face type: Inferred from calculation using, in whole or part, data from another fly type.

(1) Proportion of particles recovered (Text S1D)

$$\mathit{0.034} = \frac{\text{Number of microspheres recovered from feces (Axenic } \textit{Passaged} \text{ fly)}}{\textit{Number of microspheres ingested}}$$

$$\textit{Number of microspheres ingested} = \textit{Number of cells ingested} \times \frac{\text{Number of microspheres in inoculum}}{\text{Number of cells in inoculum}}$$

$$\textit{Number of cells ingested} = \textit{Number of CFU ingested (Axenic Immediate fly)} \times \text{Conversion factor (Text S1E)}$$

Proportion of particles recovered is calculated using samples from same fly type: Axenic *Passaged* and Axenic *Immediate* samples. Once the proportion is calculated, however, the same proportion is also applied to Gnotobiotic *Passaged* flies in the same replicate experiment.

(2) Proportion of ingested bacteria that is egested (Text S1C)

$$0.10 = \frac{\text{(Number of cells egested) / (Number of microspheres egested)}}{\text{(Number of cells ingested) / (Number of microspheres ingested)}}$$

$$\frac{\text{Number of cells egested}}{\text{Number of microspheres egested}} = \frac{\text{Number of cells recovered}}{\text{Number of microspheres recovered}}$$

$$\frac{\text{Number of cells ingested}}{\text{Number of microspheres ingested}} = \frac{\text{Number of cells in inoculum}}{\text{Number of microspheres in inoculum}}$$

(3) Proportion of ingested bacteria that is retained (Text S1C)

$$\mathbf{0.03} = \frac{\text{Number of intact cells in } \textit{Passaged} \text{ fly}}{\textbf{Number of cells ingested}}$$

Number of intact cells in *Passaged* fly = Number of cfu's in *Passaged* fly × Conversion factor (Text S1E)

$$\textbf{Number of cells ingested} = \textbf{Number of microspheres ingested} \times \frac{\text{Number of cells in inoculum}}{\text{Number of microspheres in inoculum}}$$

$$\textbf{Number of microspheres ingested} = \frac{\text{Number of microspheres recovered from feces (Axenic and Gnotobiotic } \textit{Passaged} \text{ flies)}}{\textbf{Proportion of particles recovered (Axenic Immediate and Passaged flies)}}$$

Calculating the proportion of ingested bacteria that is retained involves the number of cells ingested. As above, we use the proportion of particles recovered to calculate the number of cells ingested. Proportion of particles recovered is calculated from data on Axenic flies. We assume that the proportion of particles recovered is the same within a replicate experiment for both Axenic and Gnotobiotic fecal samples. Therefore, calculating the proportion of ingested bacteria that is retained for Axenic flies would only involve data from the same fly type. The calculation for Gnotobiotic flies, however, would involve data from another fly type.

(4) Proportion of ingested bacteria that is lysed (Text S1C)

$$\mathbf{0.87} = 1 - \text{Proportion of ingested bacteria that is egested} - \textbf{Proportion of ingested bacteria that is retained}$$

The proportion of ingested bacteria that is retained in Axenic *Passaged* samples would only involve data from the same fly type, whereas the proportion of ingested bacteria that is retained in Gnotobiotic *Passaged* samples would involve data from another fly type.

References

1. **Marx CJ**, **Lidstrom ME**. 2001. Development of improved versatile broad-host-range vectors for use in methylotrophs and other Gram-negative bacteria. Microbiology **147**:2065–2075.

2. **Shanks RMQ**, **Caiazza NC**, **Hinsa SM**, **Toutain CM**, **O'Toole GA**. 2006. *Saccharomyces cerevisiae*-based molecular tool kit for manipulation of genes from Gram-negative bacteria. Appl Environ Microbiol **72**:5027–5036.

3. **Deeraksa A**, **Moonmangmee S**, **Toyama H**, **Yamada M**, **Adachi O**, **Matsushita K**. 2005. Characterization and spontaneous mutation of a novel gene, *polE*, involved in pellicle formation in *Acetobacter tropicalis* SKU1100. Microbiology **151**:4111–4120.

4. **Carpenter AE**, **Jones TR**, **Lamprecht MR**, **Clarke C**, **Kang IH**, **Friman O**, **Guertin DA**, **Chang JH**, **Lindquist RA**, **Moffat J**, **Golland P**, **Sabatini DM**. 2006. CellProfiler: image analysis software for identifying and quantifying cell phenotypes. Genome Biol **7**:R100.
