# Supplementary figures and images for: Spatiotemporally Heterogeneous Population Dynamics of Gut Bacteria Inferred from Fecal Time Series Data
Source: mBio. 2018 Jan 9;9(1):e01453-17. doi: 10.1128/mBio.01453-17 (PMC5760738; doi:10.1128/mBio.01453-17)

CFU/ml without tetracycline

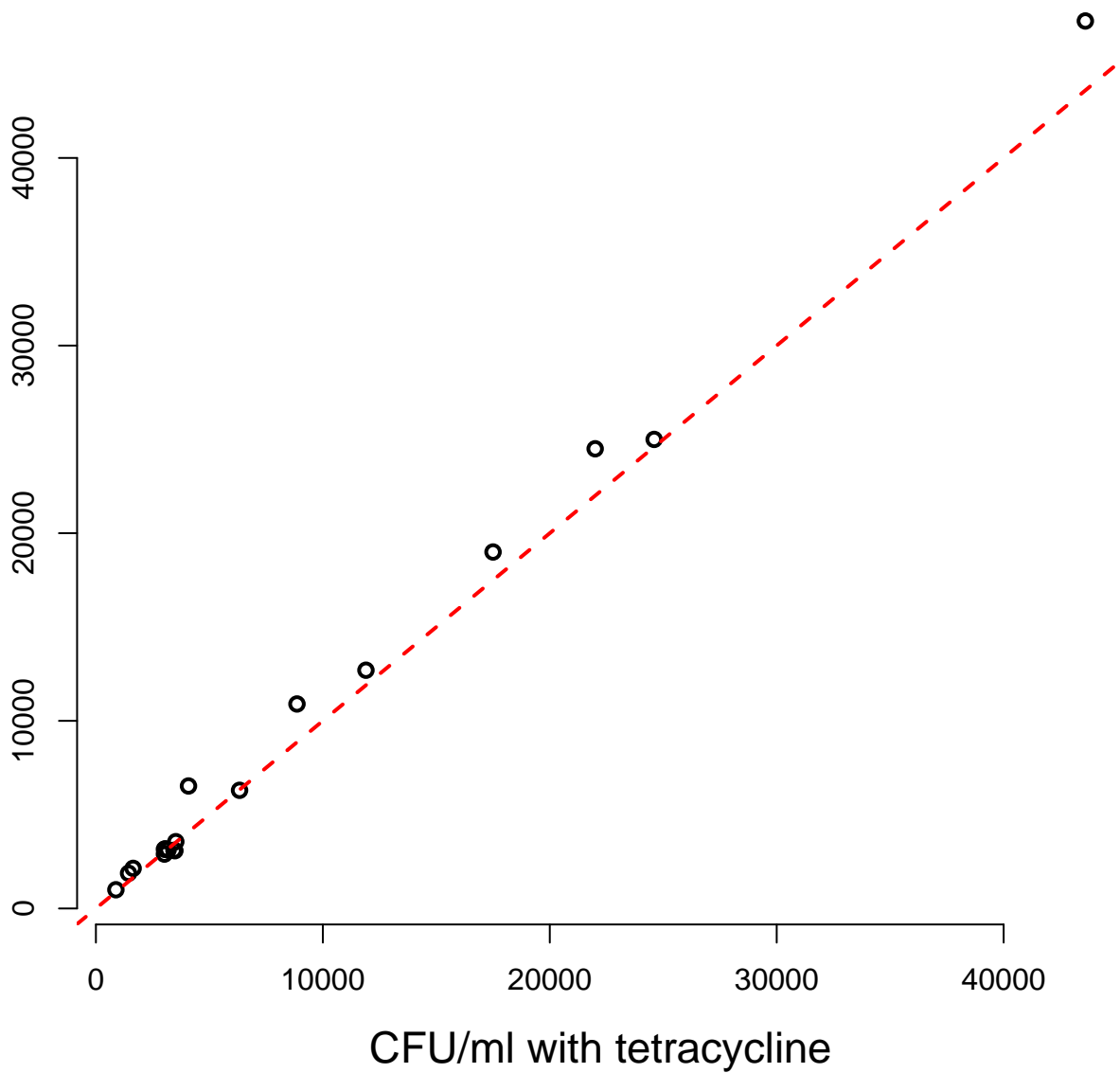

CFU/ml with tetracycline

Supplement: FIG S1 [file mbo001183662sf1.pdf]

# Microsphere

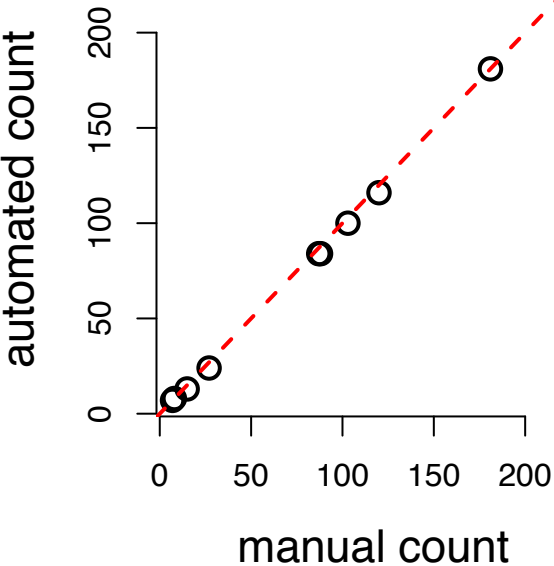

# Bacteria

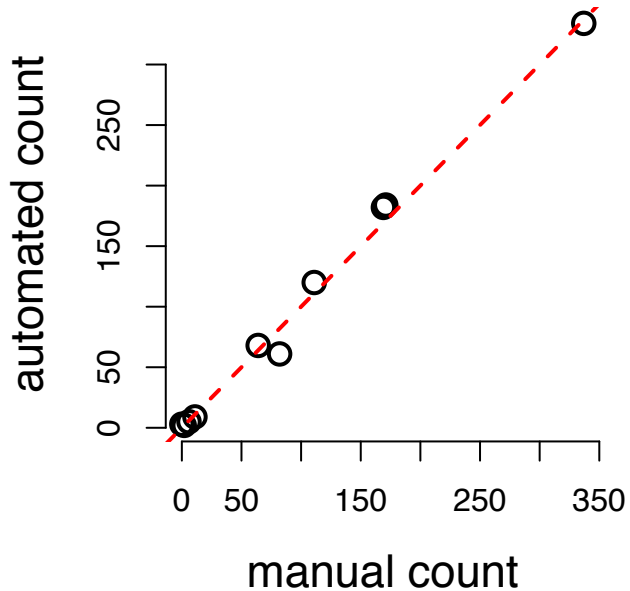

Supplement: FIG S2 [file mbo001183662sf2.pdf]

(A)

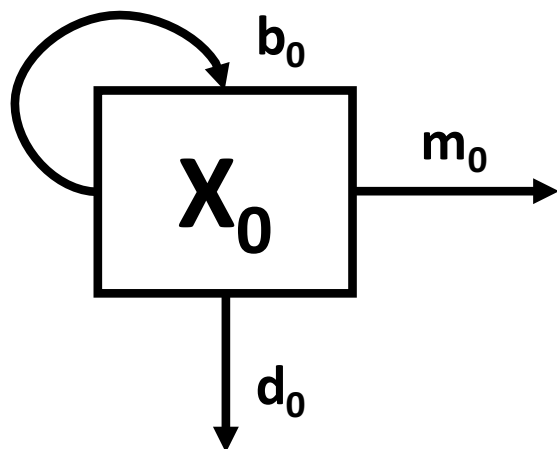

(B)

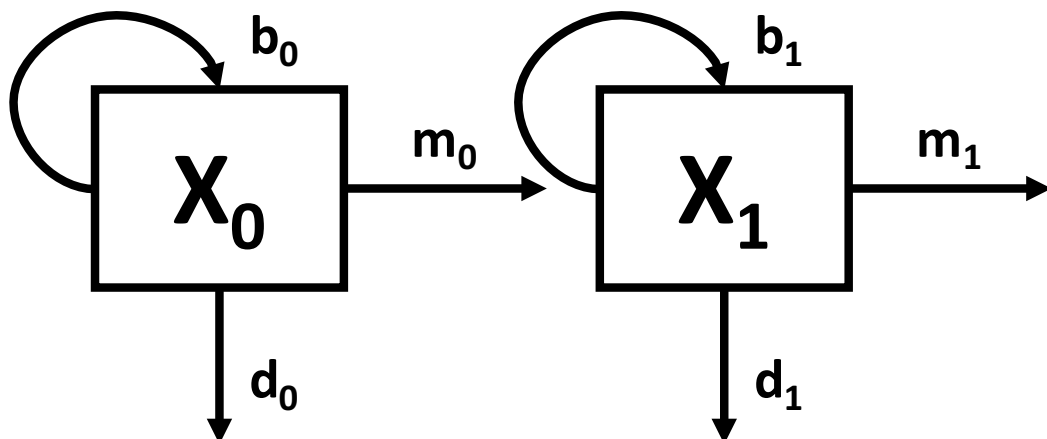

(C)

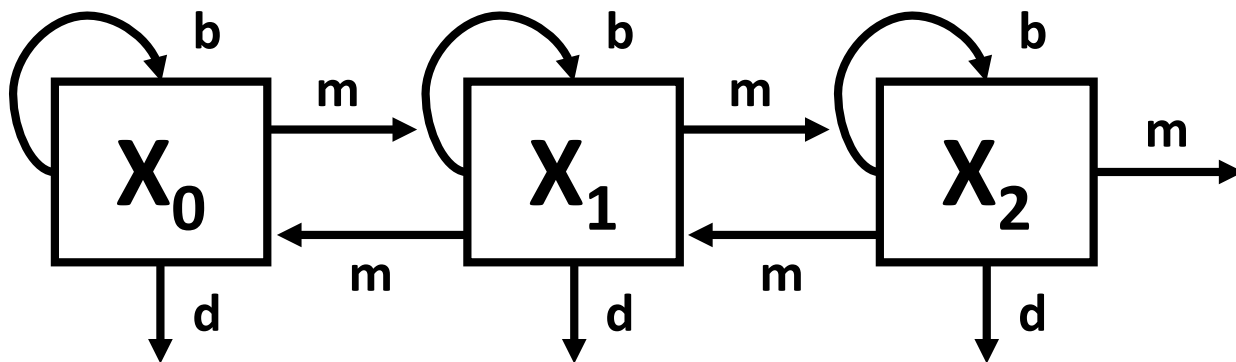

Supplement: FIG S3 [file mbo001183662sf3.pdf]

Some examples of  $f$

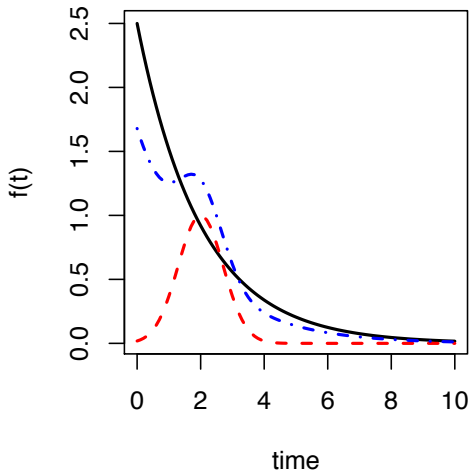

Some examples of  $g$

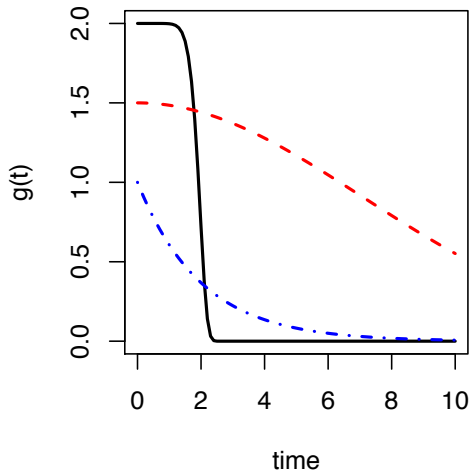

Supplement: FIG S5 [file mbo001183662sf5.pdf]

## Theory

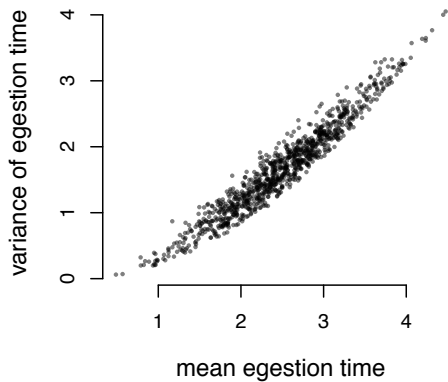

## Experiment

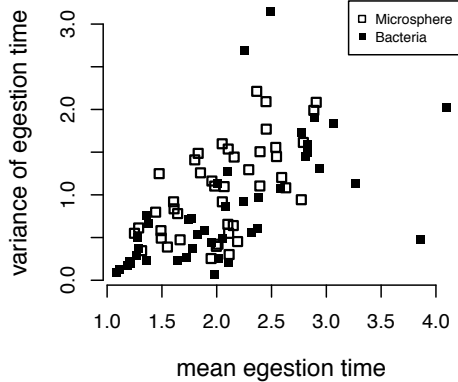

## Theory

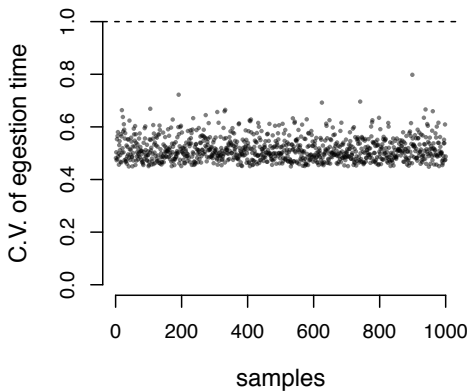

## Experiment

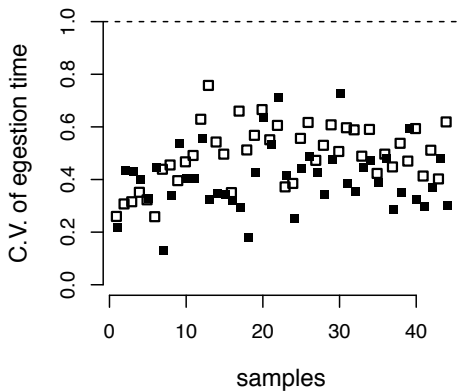

Supplement: FIG S6 [file mbo001183662sf6.pdf]
