# Supplementary material for: Spatiotemporally Heterogeneous Population Dynamics of Gut Bacteria Inferred from Fecal Time Series Data
Source: mBio. 2018 Jan 9;9(1):e01453-17. doi: 10.1128/mBio.01453-17 (PMC5760738; doi:10.1128/mBio.01453-17)
Supplement: TEXT S2 [file mbo001183662s2.pdf]

Text S2: Derivation of theoretical egestion time statistics from models

Inamine et al., for *mBio*

Here, we derive egestion time statistics in simple models that allow us to infer ecological processes affecting microbial population dynamics in the host. While it is difficult to directly measure population dynamics *in situ* (i.e. track population size of the microorganisms directly in the host gut over time), we show that ecological processes can be inferred by observing some statistical properties of the egestion time.

We first define some of the relevant terms:

- **Compartment** refers to a microbial population in a region along the gut with more or less the same characterization. For example, separating bacteria into populations in foregut, midgut, and hindgut corresponds to a system with 3 compartments.
- **Egestion time** refers to the time that a particle (*i.e.* a microorganism or a microsphere) is egested from the last compartment. Here, we treat it as a random variable.
- **Apparent death** refers to a microorganism disappearing from a compartment, instead of getting egested. Because egestion time is recorded only when a microorganism successfully exits the host, a "dead" microorganism does not have an egestion time. Apparent death could be caused by actual death (e.g. due to aging, immunity), or could be due to retention of a microorganism by the host (e.g. long-term adhesion to the host gut).

Text S2A and B are for mathematically-inclined readers. Text S2A derives the formulae for the mean and the variance of the egestion time, and intuition behind our results is further confirmed in Text S2B. In Text S2C.1, we use data from *Egestion Time Experiment* (see Main Text) to test our models. The biological implications of all the theoretical work is presented in Text S2C.2 and C.3. In summary, any ecological processes contributing to apparent death leads to shorter mean and variance of the egestion time. Egestion time statistics and proportion of ingested microorganisms that is egested together allow us to infer within-host population dynamics from fecal time series data.

A Compartment models

A.1 One compartment model

We start with the simplest model, assuming a single, well-mixed, homogenous gut. Assume that there are some initial number of microorganism N in the gut, the net population growth rate per microorganism is $r_0 = b_0 - d_0$, and the emigration rate per microorganism is m_0 (Fig. S3A). $X_0(t)$ represents the number of microorganism in the gut at time t, so $X_0(t = 0) = N$. Further assume that $r_0 < m_0$, so $r_0 - m_0 < 0$. Otherwise, $X_0(t)$ increases to $+\infty$, contrary to our observation (see Fig. 1 and 3B). Note that if $b_0 = 0$ but $d_0 > 0$ (i.e. no birth, but some microorganisms die or are retained), then $r_0 < 0$ and our assumption holds. Under these assumptions, gut microbial population size changes as:

$$\frac{dX_0}{dt} = (r_0 - m_0)X_0 \tag{S2.1}$$

The solution to this system is $X_0(t) = Ne^{(r_0-m_0)t}$. With the emigration rate m_0 and the microbial population size $X_0(t)$, the number of microorganisms egested at time t is $m_0X_0(t)$ and the total number of microorganisms egested over time is $\int_0^\infty m_0X_0(t)dt$. Then, the proportion of microorganisms egested at time t relative to the total microorganisms egested is $p(t) = \frac{m_0X_0(t)}{\int_0^\infty m_0X_0(t)dt}$. Let $\hat{t}_0$ be an egestion time of a microorganism, so $E[\hat{t}_0]$ and $Var[\hat{t}_0]$ are the mean and variance of the egestion time, respectively. For convenience, define $q_0 = r_0 - m_0 < 0$. Then,

$$\begin{aligned} E[\hat{t}_0] &= \int_0^\infty tp(t)dt = \int_0^\infty t\frac{m_0X_0}{\int_0^\infty m_0X_0dt}dt = \frac{\int_0^\infty tX_0dt}{\int_0^\infty X_0dt} = \frac{\int_0^\infty te^{q_0t}dt}{\int_0^\infty e^{q_0t}dt} = \frac{1/(r_0-m_0)^2}{-1/(r_0-m_0)} \\ &= \frac{1}{m_0 - r_0} \end{aligned} \tag{S2.2}$$

$$
\begin{aligned}
Var[\hat{t_0}] = E[\hat{t_0^2}] - E[\hat{t_0}]^2 &= \int_0^\infty t^2 p(t)dt - \left(\frac{1}{m_0 - r_0}\right)^2 = \frac{\int_0^\infty t^2 e^{q_0 t}dt}{\int_0^\infty e^{q_0 t}dt} - \frac{1}{q_0^2} = \frac{-2/q_0^3}{-1/q_0} - \frac{1}{q_0^2} \\
&= \frac{2}{q_0^2} - \frac{1}{q_0^2} = \frac{1}{(m_0 - r_0)^2}
\end{aligned} \tag{S2.3}
$$

In particular, $E[\hat{t_0}] = \frac{1}{m_0}$ and $Var[\hat{t_0}] = \frac{1}{m_0^2}$ when $r_0 = 0$.

A.2 Two compartments model

A more realistic model would have at least two compartments. For example, food is first stored in fly crop, and slowly enters the gut over time. Given the different anatomical and physiological environments in these two compartments, microorganisms may encounter different immune responses and niche availability. In our experiment, flies were starved for 4 hrs to clear out the gut. We then fed the flies for an hour, so the ingested bacteria is more dense in fly anterior than the posterior.

Assume there are some initial number of bacteria N in the source compartment (*e.g.* crop, food in the environment, *etc.*) and the microorganisms flow into the egesting compartment (*e.g.* gut) over time (Fig. S3B). The net population growth rate per microorganism in compartment i is $r_i = b_i - d_i$, where $i = 0$ or 1 corresponds to the source and egesting compartment, respectively. Similarly, the emigration rate per microorganism is m_i and $X_i(t)$ is the number of microorganism at time t. Assume that $r_i < m_i$, so $q_i = r_i - m_i < 0$ for all i. Under these assumptions, gut microbial population sizes change as:

$$
\begin{aligned}
\frac{dX_0}{dt} &= q_0 X_0 \\
\frac{dX_1}{dt} &= m_0 X_0 + q_1 X_1
\end{aligned} \tag{S2.4}
$$

The solution to this system is $X_0(t) = Ne^{q_0 t}$ and $X_1(t) = \frac{m_0 N}{q_0 - q_1}(e^{q_0 t} - e^{q_1 t})$ assuming $q_0 \neq q_1$ (the final result will be the same even if $q_0 = q_1$). Let $\hat{t_1}$ be the egestion time from the egesting compartment, so $E[\hat{t_1}]$ and $Var[\hat{t_1}]$ are the mean and variance of the egestion time, respectively. Then,

$$
\begin{aligned}
E[\hat{t_1}] &= \int_0^\infty t p(t)dt = \frac{\int_0^\infty tX_1 dt}{\int_0^\infty X_1 dt} = \frac{\int_0^\infty t(e^{q_0 t} - e^{q_1 t})dt}{\int_0^\infty e^{q_0 t} - e^{q_1 t}dt} = \frac{q_1^2 - q_0^2}{q_0^2 q_1^2}\frac{q_1 q_0}{q_0 - q_1} = \frac{-q_1 - q_0}{q_1 q_0} \\
&= -\frac{1}{q_0} - \frac{1}{q_1}
\end{aligned} \tag{S2.5}
$$

$$
\begin{aligned}
Var[\hat{t_1}] = E[\hat{t_1^2}] - E[\hat{t_1}]^2 &= \int_0^\infty t^2 p(t)dt - (\frac{1}{q_0} + \frac{1}{q_1})^2 = \frac{\int_0^\infty t^2(e^{q_0 t} - e^{q_1 t})dt}{\int_0^\infty e^{q_0 t} - e^{q_1 t}dt} - (\frac{1}{q_0} + \frac{1}{q_1})^2 \\
&= \frac{2}{q_0^2 q_1^2}\frac{q_0^3 - q_1^3}{q_0 - q_1} - (\frac{1}{q_0} + \frac{1}{q_1})^2 = \frac{2(q_0^2 + q_0 q_1 + q_1^2)}{q_0^2 q_1^2} - \frac{(q_0 + q_1)^2}{q_0^2 q_1^2} \\
&= \frac{1}{q_0^2} + \frac{1}{q_1^2}
\end{aligned} \tag{S2.6}
$$

Note that $E[\hat{t_1}] > 0$ and specifically, $E[\hat{t_1}] = \frac{1}{m_0} + \frac{1}{m_1}$ and $Var[\hat{t_1}] = \frac{1}{m_0^2} + \frac{1}{m_1^2}$when $r_0 = r_1 = 0$.

A.3 $n + 1$ compartments model

Let us generalize the models in Text S2A.1 and A.2, by having many compartments between the source and the egesting compartments (Fig. 4A). For example, microorganisms may go through different environments in the crop, foregut, midgut, and hindgut. The net population growth rate per microorganism in compartment i is $r_i = b_i - d_i$ and the emigration rate per microorganism is m_i, where $i = 0, 1, 2, \ldots, n$. Define $q_i = r_i - m_i$. Under these assumptions, gut microbial population sizes change as:

$$
\begin{aligned}
\frac{dX_0}{dt} &= q_0 X_0 \\
\frac{dX_1}{dt} &= m_0 X_0 + q_1 X_1 \\
\frac{dX_2}{dt} &= m_1 X_1 + q_2 X_2 \\
&\vdots \\
\frac{dX_n}{dt} &= m_{n-1} X_{n-1} + q_n X_n
\end{aligned}
\tag{S2.7}
$$

Theorem 1. *Let $E[\hat{t}_i]$ and $Var[\hat{t}_i]$ be the mean and variance of a random variable $\hat{t}_i$, an egestion time from compartment X_i. Assume that $X_0(0) = N$ but $X_k(0) = 0$ for all $k = 1, 2, \dots n$, and $r_i < m_i$ so that $q_i = r_i - m_i < 0$ for all $i = 0, 1, 2, \dots n$. Then,*

$$
\begin{aligned}
E[\hat{t}_i] &= \begin{cases} -\frac{1}{q_0} & \text{if } i = 0 \\ E[\hat{t}_{i-1}] - \frac{1}{q_i} & \text{if } i > 0 \end{cases} \\
Var[\hat{t}_i] &= \begin{cases} \frac{1}{q_0^2} & \text{if } i = 0 \\ Var[\hat{t}_{i-1}] + \frac{1}{q_i^2} & \text{if } i > 0 \end{cases}
\end{aligned}
\tag{S2.8}
$$

Therefore, $E[\hat{t}_n] = \sum_{i=0}^{n} \frac{1}{m_i - r_i}$ and $Var[\hat{t}_n] = \sum_{i=0}^{n} \frac{1}{(m_i - r_i)^2}$ for any $n = 0, 1, 2, \dots$

Proof. 1. From Eqs. S2.2 and S2.5, $E[\hat{t}_0] = \frac{1}{m_0 - r_0} = -\frac{1}{q_0}$ and $E[\hat{t}_1] = -\frac{1}{q_0} - \frac{1}{q_1} = E[\hat{t}_0] - \frac{1}{q_1}$. From Eqs. S2.3 and S2.6, $Var[\hat{t}_0] = \frac{1}{q_0^2}$ and $Var[\hat{t}_1] = \frac{1}{q_0^2} + \frac{1}{q_1^2} = Var[\hat{t}_0] + \frac{1}{q_1^2}$. So, the theorem holds for $i = 0, 1$.

2. By hypothesis, $X_k(0) = 0$ for all $k = 1, 2, \dots n$. So for an arbitrary $n > 0$, the solution for $\frac{dX_n}{dt}$ is $X_n(t) = \int_0^t m_{n-1} X_{n-1}(s) e^{q_n(t-s)} ds$. Then,

$$
E[\hat{t}_n] = \frac{\int_0^\infty t X_n(t) dt}{\int_0^\infty X_n(t) dt} = \frac{\int_{t=0}^\infty \int_{s=0}^t t X_{n-1}(s) e^{q_n(t-s)} ds\, dt}{\int_{t=0}^\infty \int_{s=0}^t X_{n-1}(s) e^{q_n(t-s)} ds\, dt}
\tag{S2.9}
$$

Switch the order of integration and pull out some terms:

$$
E[\hat{t}_n] = \frac{\int_{s=0}^\infty X_{n-1}(s) e^{-q_n s} \int_{t=s}^\infty t e^{q_n t} dt\, ds}{\int_{s=0}^\infty X_{n-1}(s) e^{-q_n s} \int_{t=s}^\infty e^{q_n t} dt\, ds}
\tag{S2.10}
$$

Calculate the inner integrals in the numerator and the denominator first:

$$
\begin{aligned}
E[\hat{t}_n] &= \frac{\int_{s=0}^\infty X_{n-1}(s) e^{-q_n s} (1/q_n^2 - s/q_n) e^{q_n s}\, ds}{\int_{s=0}^\infty X_{n-1}(s) e^{-q_n s} (-e^{q_n s}/q_n)\, ds} = \frac{\int_{s=0}^\infty X_{n-1}(s) (1/q_n^2 - s/q_n)\, ds}{(-1/q_n) \int_{s=0}^\infty X_{n-1}(s)\, ds} \\
&= \frac{\frac{1}{q_n^2} \int_0^\infty X_{n-1}(s) ds - \frac{1}{q_n} \int_0^\infty s X_{n-1}(s) ds}{-\frac{1}{q_n} \int_0^\infty X_{n-1}(s) ds} = \frac{\int_0^\infty s X_{n-1}(s) ds}{\int_0^\infty X_{n-1}(s) ds} - \frac{1}{q_n} \\
&= E[\hat{t}_{n-1}] - \frac{1}{q_n}
\end{aligned}
\tag{S2.11}
$$

because $\frac{\int_0^\infty s X_{n-1}(s) ds}{\int_0^\infty X_{n-1}(s) ds} = E[\hat{t}_{n-1}]$. Next,

$$
Var[\hat{t}_n] = E[\hat{t}_n^2] - E[\hat{t}_n]^2 = \frac{\int_0^\infty t^2 X_n dt}{\int_0^\infty X_n dt} - E[\hat{t}_n]^2 = \frac{\int_{t=0}^\infty \int_{s=0}^t t^2 X_{n-1}(s) e^{q_n(t-s)} ds\, dt}{\int_{t=0}^\infty \int_{s=0}^t X_{n-1}(s) e^{q_n(t-s)} ds\, dt} - E[\hat{t}_n]^2
\tag{S2.12}
$$

Switch the order of integration, and pull out some terms. Also note that we calculated the denominator while deriving $E[\hat{t}_n]$ in Eqs. S2.9 - S2.11:

$$
Var[\hat{t}_n] = \frac{\int_{s=0}^\infty X_{n-1}(s) e^{-q_n s} \int_{t=s}^\infty t^2 e^{q_n t} dt\, ds}{(-1/q_n) \int_{s=0}^\infty X_{n-1}(s)\, ds} - E[\hat{t}_n]^2
\tag{S2.13}
$$

Calculate the inner integrals in the numerator first:

$$
\begin{aligned}
Var[\hat{t}_n] &= \frac{\int_{s=0}^{\infty} X_{n-1}(s) e^{-q_n s}(-e^{q_n s}(s^2/q_n - 2s/q_n^2 + 2/q_n^3))\, ds}{(-1/q_n)\int_{s=0}^{\infty} X_{n-1}(s)\, ds} - E[\hat{t}_n]^2 \\
&= \frac{-\frac{1}{q_n}\int_0^{\infty} s^2 X_{n-1}(s) ds + \frac{2}{q_n^2}\int_0^{\infty} s X_{n-1}(s) ds - \frac{2}{q_n^3}\int_0^{\infty} X_{n-1}(s) ds}{\frac{-1}{q_n}\int_{s=0}^{\infty} X_{n-1}(s)\, ds} - E[\hat{t}_n]^2 \\
&= \frac{\int_0^{\infty} s^2 X_{n-1}(s) ds}{\int_0^{\infty} X_{n-1}(s) ds} - \frac{2}{q_n}\frac{\int_0^{\infty} s X_{n-1}(s) ds}{\int_0^{\infty} X_{n-1}(s) ds} + \frac{2}{q_n^2} - E[\hat{t}_n]^2
\end{aligned}
\tag{S2.14}
$$

Note that $\frac{\int_0^{\infty} s^2 X_{n-1}(s) ds}{\int_0^{\infty} X_{n-1}(s) ds} = E[\hat{t}_{n-1}^2]$ and $\frac{\int_0^{\infty} s X_{n-1}(s) ds}{\int_0^{\infty} X_{n-1}(s) ds} = E[\hat{t}_{n-1}]$:

$$
Var[\hat{t}_n] = E[\hat{t}_{n-1}^2] - \frac{2}{q_n} E[\hat{t}_{n-1}] + \frac{2}{q_n^2} - E[\hat{t}_n]^2 \tag{S2.15}
$$

Finally, using $E[\hat{t}_n] = E[\hat{t}_{n-1}] - \frac{1}{q_n}$ dervied in Eq. S2.11:

$$
\begin{aligned}
Var[\hat{t}_n] &= E[\hat{t}_{n-1}^2] - \frac{2}{q_n} E[\hat{t}_{n-1}] + \frac{2}{q_n^2} - \left(E[\hat{t}_{n-1}] - \frac{1}{q_n}\right)^2 \\
&= E[\hat{t}_{n-1}^2] - E[\hat{t}_{n-1}]^2 - \frac{2}{q_n} E[\hat{t}_{n-1}] + \frac{2}{q_n} E[\hat{t}_{n-1}] + \frac{2}{q_n^2} - \frac{1}{q_n^2} \\
&= Var[\hat{t}_{n-1}] + \frac{1}{q_n^2}
\end{aligned}
\tag{S2.16}
$$

by noticing that $E[\hat{t}_{n-1}^2] - E[\hat{t}_{n-1}]^2 = Var[\hat{t}_{n-1}]$

3. By induction, $E[\hat{t}_0] = -\frac{1}{q_0}$ and $E[\hat{t}_i] = E[\hat{t}_{i-1}] - \frac{1}{q_n}$ for all $i = 1, 2, \ldots$ Similarly, $Var[\hat{t}_0] = \frac{1}{q_0^2}$ and $Var[\hat{t}_i] = Var[\hat{t}_{i-1}] + \frac{1}{q_n^2}$ for all $i = 1, 2, \ldots$ It follows that $E[\hat{t}_n] = \sum_{i=0}^{n} -\frac{1}{q_i} = \sum_{i=0}^{n} \frac{1}{m_i - r_i}$ and $Var[\hat{t}_n] = \sum_{i=0}^{n} \frac{1}{q_i^2} = \sum_{i=0}^{n} \frac{1}{(m_i - r_i)^2}$ for all $n = 0, 1, 2, 3, \ldots$

□

In particular, $E[\hat{t}_n] = \sum_{i=0}^{n} \frac{1}{m_i}$ and $Var[\hat{t}_n] = \sum_{i=0}^{n} \frac{1}{m_i^2}$ if $r_i = 0$ for all $i = 0, 1, 2, \ldots$.

A.4 Numerical exploration of bi-directional migration

The biological intuition behind our results above is simple and straightforward: as death/retention rate in the host increases (*i.e.* $r = b - d$ decreases), microorganisms that are observed to egest are biased towards earlier egestion time. Longer egestion time means that a microorganism must endure higher probability of death/retention, and therefore lowers the probability of it being detected in an experiment. Furthermore, as this detection bias gets stronger, we expect less variance. The same logic applies to higher emigration rate (*i.e.* higher m).

However, does the same intuition work when we relax our model assumptions? For example, we assumed that the microorganism can only flow unidirectionally through the compartments. While this is often a valid assumption, it may not always be the case (*e.g.* regurgitation of food from the crop). We now relax this assumption and consider an extreme case: a microorganism has an equal probability of migrating to the posterior compartment as well as the anterior compartment (Fig. S3C). Specifically, we simulated the following model:

$$
\begin{aligned}
\frac{dX_0}{dt} &= (r - m)X_0 + mX_1 \\
\frac{dX_1}{dt} &= (r - 2m)X_1 + mX_0 + mX_2 \\
\frac{dX_2}{dt} &= (r - 2m)X_2 + mX_1
\end{aligned}
\tag{S2.17}
$$

Even under this assumption, we observed that decreasing r always leads to decreasing mean egestion time. Bidirectional emigration therefore does not change the qualitative effect of r on the mean egestion time. We also

observed a pattern in variance consistent with our previous analyses. (Fig. S4). Similarly, we observed that increasing m always leads to decreasing mean egestion time. Bidirectional emigration therefore does not change the qualitative effect of m on the mean egestion time. Again, we observed a pattern in variance consistent with our previous analyses. (Fig. S4).

Importantly, we observed that the distributions of the egestion time in our simulations are **stochastically ordered**; that is, the cumulative distribution curve of a larger r (smaller m) always lie on or under the curve of a smaller r (larger m). Stochastic ordering is consistent with our intuition, as any ecological process biasing the distribution towards earlier egestion time would lead to stochastically smaller distribution. In Section B, we analyze larger class of models to show that our result in stochastic ordering does not hinge on our specific model structure.

B Structural model

Our results from Text S2A imply that, all else being equal, apparent death of the microorganisms leads to stochastically smaller distribution. In this section, we test whether apparent death alone could lead to stochastic ordering, without hinging on specific model structure. To do so, we drop the assumption of compartments and ignore the specific movement pattern of the microorganisms within the gut. Instead, we focus on the distribution of microorganisms egested over time with and without apparent death. The basic idea is as follows. We track the microorganisms as they move through the gut. We "mark" them as dead when they die, but we let them continue to move as if they were still alive, and count them when they exit. We then have two different distributions stemming from the exact same egestion pattern: a distribution of microorganisms with apparent death (only unmarked microorganisms) and without apparent death (both marked and unmarked microorganisms). We compare these two distributions to understand the effect of apparent death.

We use the function $f(t)$ to represent the number of microorganisms egested at time t if apparent death were absent. We use the function $f(t)g(t)$ to describe the number of unmarked microorganisms egested if apparent death were present. The function $g(t)$ is survival rate. It decreases monotonically with time, because the longer a microorganism stays in the host, more likely it would be marked as dead. The assumption that $g' \leq 0$ is natural for a mortality-only situations, because it just says that the longer a microorganism spends in the host, the more likely it is to be marked dead. Our model below is actually more general than mortality-only situations, and Theorem 2 is true even if $g(t)$ is the net result of reproduction and mortality. In fact, a microorganism can even have positive net reproduction rate in the gut, as long as total amount of f over time eventually goes to 0.

In terms of Text S2A, f is the number of microorganisms we obtain if we set $r_i = 0$ for all compartments i. Conversely, fg is the number of microorganisms we obtain if we have $r_i = b_i - d_i < 0$ for any compartment i. Without specifying the exact functions, we ask if qualitative assumptions below are sufficient to generate a bias in mean egestion time. See Fig. S5 for some examples of f and g satisfying the assumptions below.

Theorem 2. *Let $f(t)$ be the number of microorganisms egested at time t, and $g(t)$ be the survival rate of the microorganisms egested at time t. Assume that f and g are both Lebesgue integrable and continuously differentiable, $f, g \geq 0$ for all t, f is bounded and $g'(t) \leq 0$ for all t. Let t_0 and t_1 be the egestion time random variable for the microorganisms without and with apparent death, respectively. Then,*

$$P(t_0 \leq a) \equiv \int_0^a \frac{f(t)}{\int_0^\infty f(x)dx}dt \leq \int_0^a \frac{f(t)g(t)}{\int_0^\infty f(x)g(x)dx}dt \equiv P(t_1 \leq a) \tag{S2.18}$$

*for all $a \geq 0$, i.e. t_0 is **stochastically greater** (written $\geq_{st}$) than t_1.*

Proof. First, note that both denominators are positive constants in the inequality above:

$$\begin{aligned} \int_0^a \frac{f(t)}{\int_0^\infty f(x)dx}dt \leq \int_0^a \frac{f(t)g(t)}{\int_0^\infty f(x)g(x)dx}dt &\iff \frac{\int_0^a f(t)dt}{\int_0^\infty f(t)dt} \leq \frac{\int_0^a f(t)g(t)dt}{\int_0^\infty f(t)g(t)dt} \\ &\iff \int_0^a f(t)dt \int_0^\infty f(t)g(t)dt \leq \int_0^a f(t)g(t)dt \int_0^\infty f(t)dt \end{aligned} \tag{S2.19}$$

Second, for a fixed a, let $H_a(x) \equiv \int_0^a f(t)dt \int_0^x f(t)g(t)dt$ and $J_a(x) \equiv \int_0^a f(t)g(t)dt \int_0^x f(t)dt$:

$$\begin{aligned}\int_0^a f(t)dt \int_0^\infty f(t)g(t)dt \leq \int_0^a f(t)g(t)dt \int_0^\infty f(t)dt &\iff \lim_{x\to\infty} H_a(x) \leq \lim_{x\to\infty} J_a(x) \\ &\iff \lim_{x\to\infty} \int_0^x H_a'(y)dy \leq \lim_{x\to\infty} \int_0^x J_a'(y)dy \\ &\iff \lim_{x\to\infty} \left[\int_0^a H_a'(y)dy + \int_a^x H_a'(y)dy\right] \leq \lim_{x\to\infty} \left[\int_0^a J_a'(y)dy + \int_a^x J_a'(y)dy\right]\end{aligned} \tag{S2.20}$$

Third, note that $H_a(a) = J_a(a)$:

$$\begin{aligned}\lim_{x\to\infty} \left[\int_0^a H_a'(y)dy + \int_a^x H_a'(y)dy\right] &\leq \lim_{x\to\infty} \left[\int_0^a J_a'(y)dy + \int_a^x J_a'(y)dy\right] \\ \iff \lim_{x\to\infty} \int_a^x H_a'(y)dy &\leq \lim_{x\to\infty} \int_a^x J_a'(y)dy\end{aligned} \tag{S2.21}$$

To prove the inequality in Eq. (S2.21) for all a, we shall consider the derivatives of H_a and J_a with respect to x.

$$\begin{aligned}H_a'(x) &= \left[\int_0^a f(t)dt\right] f(x)g(x) \\ J_a'(x) &= \left[\int_0^a f(t)g(t)dt\right] f(x)\end{aligned} \tag{S2.22}$$

By hypothesis, $g' \leq 0$ which implies that $\int_0^a f(t)g(t)dt \geq [\int_0^a f(t)dt]g(a)$. So,

$$H_a'(y) = \left[\int_0^a f(t)dt\right] f(y)g(y) \leq \left[\int_0^a f(t)dt\right] f(y)g(a) \leq \left[\int_0^a f(t)g(t)dt\right] f(y) = J_a'(y) \tag{S2.23}$$

for $y \geq a$. Finally, H_a' and J_a' are positive and integrable, so $H_a' \leq J_a'$ for $y \geq a$ implies

$$\int_a^x H_a'(y)dy \leq \int_a^x J_a'(y)dy \implies \lim_{x\to\infty} \int_a^x H_a'(y)dy \leq \lim_{x\to\infty} \int_a^x J_a'(y)dy \tag{S2.24}$$

which holds for any chosen $a \geq 0$. □

Theorem 2 leads us immediately to some useful statistical properties:

- $t_0 \geq_{st} t_1$ implies that $E[u(t_0)] \geq E[u(t_1)]$ for any non-decreasing function u. In particular, $E[t_0] \geq E[t_1]$.
- Suppose $E[t_0] = E[t_1]$. Then, $Var[t_0] \geq Var[t_1]$.
- Suppose we want to compare mutant with wild-type microorganisms, where we have a common baseline death rate $k(t)$ for both but time-dependent benefit $g(t)$ for mutant. Suppose $k(t)$ is continuously differentiable. Then, we can replace $f(t)$ with $\hat{f}(t) = f(t)k(t)$ and Theorem 2 holds.

Conjecture 1. *Under the assumptions in Theorem 2,* $Var[t_1] \leq Var[t_0]$.

C Supporting theory with empirical data and inferring biological process from theoretical results

To apply our theory to our experiments, we first validate our theory by testing some of its key predictions using our experimental results. We then describe how we use our theoretical results to understand the processes occurring in the host.

C.1 Testing theory with experimental and simulated data

For the model in Fig. 4A, we derived the mean egestion time as $\sum_{i=0}^{n} 1/(m_i - r_i)$ and the variance of the egestion time as $\sum_{i=0}^{n} 1/(m_i - r_i)^2$, where m_i and r_i refer to emigration rate and net reproductive rate at the ith compartment. For simplicity, let $k_i = 1/(m_i - r_i)$. The mean egestion time is then $\sum_{i=0}^{n} k_i$ and the variance of the egestion time is $\sum_{i=0}^{n} k_i^2$. These formulae lead to two key predictions. We test these predictions on microsphere and microbial egestion time data from our *Egestion Time Experiment* (Main Text) and simulated data. Simulated data were generated for 1000 samples, each with 5 compartments. For each simulated sample, we drew $\{k_i\}$ from continuous uniform distribution with domain $[0, 1]$. We then calculated the mean and the variance from $\{k_i\}$ according to the formulae.

First, suppose that we have two samples, A and B. Suppose sample A has higher k_i in some compartments than in sample B, but the same k_i in the other compartments. Then, the mean (variance) of the egestion time of sample A is larger than the mean (variance) of the egestion time of sample B. Therefore, we predict positive correlation between the mean and the variance of the egestion time. To test this prediction, we performed correlation test between mean and the variance of the egestion time using Spearman's rank correlation. Both simulated and experimental data showed positive correlation (Simulated: Spearman's $\rho = 0.96$, $p < 2.2 \times 10^{-16}$; Microsphere: Spearman's $\rho = 0.57$, $p = 8 \times 10^{-5}$; Bacteria: Spearman's $\rho = 0.75$, $p = 5.7 \times 10^{-8}$; Fig. S6, top row)

Second, our formulae show that

$$\frac{Variance}{Mean^2} = \frac{\sum_{i=0}^{n} k_i^2}{(\sum_{i=0}^{n} k_i)^2} = \frac{\sum_{i=0}^{n} k_i^2}{\sum_{i=0}^{n} k_i^2 + 2\sum_{i \neq j} k_i k_j} \leq 1 \qquad \text{(S2.25)}$$

Eq. S2.25 implies that the coefficient of variation (C.V.) = $\sqrt{Var/Mean^2} \leq 1$. To test this prediction, we performed Student's t-test against the null hypothesis of $\mu = 1$. Both simulated and experimental data showed that the mean of C.V. values are significantly less than 1 (Simulated: mean C.V. = 0.52, $p < 2.2 \times 10^{-16}$; Microsphere: mean C.V. = 0.49, $p < 2.2 \times 10^{-16}$; Bacteria: mean C.V. = 0.41, $p < 2.2 \times 10^{-16}$; Fig. S6, bottom row)

To ensure that our simulated dataset were not dependent on the number of compartments or the domain of $\{k_i\}$, we also generated 1000 samples where the number of compartments was randomly generated from discrete uniform distribution with domain $[1, 50]$, and $\{k_i\}$ were drawn from continuous uniform distribution with domain $[0, 100]$. Our conclusions remained the same with this dataset.

Taken together, the data from our experiment support our theory. Next, we use our theory to further analyze our data.

C.2 Biological implications and inferring within-host population dynamics

Our theoretical results lead to important biological implications:

- The intuition behind our results is the following: as apparent death rate in the host increases (i.e. lower r), microorganisms that are egested intact are biased towards earlier egestion time whereas dead/retained microorganisms are not observed to egest. This bias towards earlier egestion time translates into lower mean of the egestion time. Furthermore, as the bias gets stronger, we expect less variance. The same logic applies to higher emigration rate (i.e. higher m).

- Even under a very general model, we showed that the qualitative results do not change; any ecological processes contributing to apparent death leads to shorter mean egestion time. Mean egestion time therefore is useful in inferring demographic processes.

- We do not need to measure the number of microorganisms ingested by the host; the time-series of the microorganisms egested is sufficient for calculating statistics of the egestion time distribution. Our approach could therefore be applied to pre-existing datasets, where the number of egested microorganisms was tracked over time.

While the number of ingested microorgansims is unnecessary to calculate egestion time statistics, it nevertheless provides additional information on the microbial demographic processes. Below, we categorize different demographic patterns of the microorganisms in the host, by leveraging both the proportion of microorganisms that is egested (relative to the number ingested) and the egestion time statistics.

C.3 Demographic interpretation of comparisons within treatments or between treatments using proportion of ingested microorganisms that is egested and egestion time statistics.

In Fig. 4B we identified four possible patterns in the fecal data resulting from different demographic processes in the host. Here we use numerical examples to illustrate this classification scheme. Table Table S1 gives parameter values describing four hypothetical microbial populations and a hypothetical microsphere "population" used in these examples. All five of these populations had the same set of m_i values, but differed in the set of r_i values. Microspheres had the same set of m_i parameter values as the microbial population (*i.e.* $m_0 = 5, m_1 = m_2 = m_3 = m_4 = 1$), but had $r_i = 0$ for all i (no birth or mortality). We use Theorem 1 (*i.e.* mean egestion time $= \sum_{i=0}^{n} \frac{1}{m_i - r_i}$ and variance of the egestion time $= \sum_{i=0}^{n} \frac{1}{(m_i - r_i)^2}$), together with the proportion of ingested particles (bacteria or microspheres) that is egested, to characterize how population dynamics are affected by the within-host processes.

Example 1: Equal proportions egested, and equal mean and variance of the egestion time.

Consider population A and microsphere in Table S1. Throughout the gut, population A has $r_i = 0$ for all i and therefore has the same population dynamics as the microspheres. Consequently, the proportion of ingested particles that is egested, the mean, and the variance of the egestion time are equal between microspheres and population A.
$\Longrightarrow$ *In general, if two types of particle have no demographic differences, we expect an equal proportion of ingested particles that is egested, and equal mean and variance of the egestion times.*

Example 2: Lower proportion egested, and lower mean and variance of the egestion time.

Consider population A and population D in Table S1. Population D has large mortality and retention occurring across multiple compartments ($r_i = -5$ for all i), resulting in only 0.04% of bacteria being egested in feces. Reduced population size in the gut affects the egestion time distribution, resulting in smaller mean and variance of the egestion time for population D than for population A.
$\Longrightarrow$ *In general, if one particle type has additional mortality and retention compared to another particle type, occurring gradually throughout gut passage, we expect a lower proportion that is egested and lower mean and variance of the egestion time.*

Example 3: Lower proportion of particles egested but equal mean and variance of the egestion time.

Consider population B and the microspheres in Table S1. In population B, mortality and retention happen quickly and intensely in one compartment, but not in others. The fraction of particles egested is lower for the bacteria, by a factor of 2. In contrast, the mean and variance of egestion time are both very similar to those for the microspheres.
$\Longrightarrow$ *In general, if mortality or retention occur rapidly, the proportion of ingested particles that is egested can be low even when the effect on the egestion time distribution is very small.*

Example 4: Equal porportion of particles egested but lower mean and variance of the egestion time.

Consider population B and population C in Table S1. In population B, mortality and retention happen quickly and intensely in one compartment. In population C, mortality and retention happen gradually but persistently across the gut. Here, the mean egestion time for population B is 4.1, and the mean egestion time for population C is 3.6. Similarly, the variance of the egestion time for population B is 4.01, and the variance of the egestion time for population C is 2.91. Despite these differences, the fractions of bacteria that survive to be egested in feces are equal (both populations 50%).
$\Longrightarrow$ *In general, gradual but persistent mortality and retention can result in a similar proportion of ingested particles that is egested as quick and intense mortality and retention, but the effect on the egestion time distribution is large. We expect similar proportion of ingested particles that is egested between the two, but large difference in the egestion time statistics.*
