# Supplementary material for: Spatiotemporally Heterogeneous Population Dynamics of Gut Bacteria Inferred from Fecal Time Series Data
Source: mBio. 2018 Jan 9;9(1):e01453-17. doi: 10.1128/mBio.01453-17 (PMC5760738; doi:10.1128/mBio.01453-17)
Supplement: FIG S4 [file mbo001183662sf4.pdf]

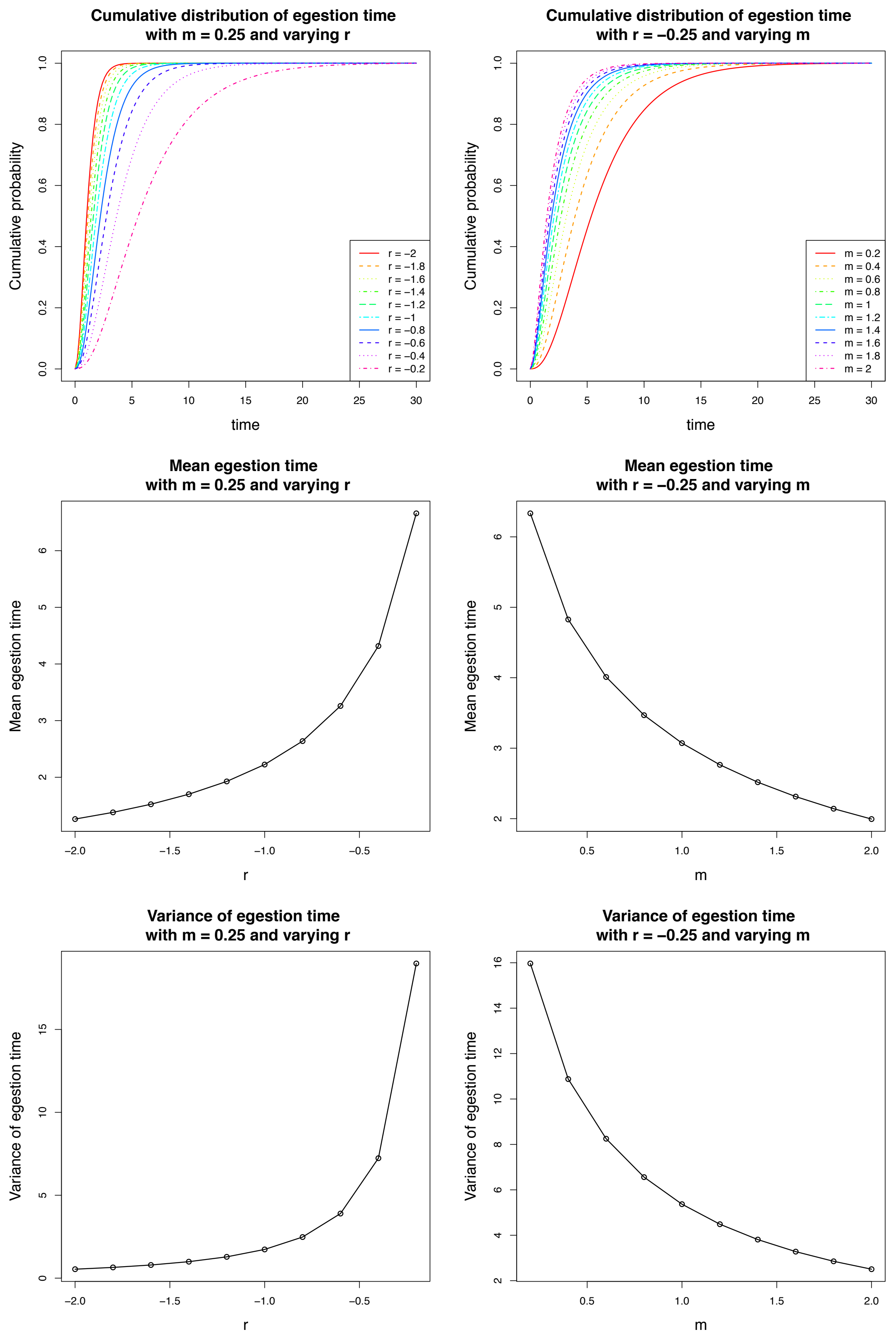

Cumulative distribution of egestion time
with m = 0.25 and varying r
Cumulative probability
time
r = −2
r = −1.8
r = −1.6
r = −1.4
r = −1.2
r = −1
r = −0.8
r = −0.6
r = −0.4
r = −0.2
Cumulative distribution of egestion time
with r = −0.25 and varying m
Cumulative probability
time
m = 0.2
m = 0.4
m = 0.6
m = 0.8
m = 1
m = 1.2
m = 1.4
m = 1.6
m = 1.8
m = 2
Mean egestion time
with m = 0.25 and varying r
Mean egestion time
r
Mean egestion time
with r = −0.25 and varying m
Mean egestion time
m
Variance of egestion time
with m = 0.25 and varying r
Variance of egestion time
r
Variance of egestion time
with r = −0.25 and varying m
Variance of egestion time
m
