# Supplementary material for: Spatiotemporally Heterogeneous Population Dynamics of Gut Bacteria Inferred from Fecal Time Series Data
Source: mBio. 2018 Jan 9;9(1):e01453-17. doi: 10.1128/mBio.01453-17 (PMC5760738; doi:10.1128/mBio.01453-17)
Supplement: TABLE S1 [file mbo001183662st1.pdf]

Population	r_0	r_1	r_2	r_3	r_4	%	μ	σ^2
Microsphere	0	0	0	0	0	100	4.2	4.04
A	0	0	0	0	0	100	4.2	4.04
B	-5	0	0	0	0	50	4.1	4.01
C	-0.18	-0.18	-0.18	-0.18	-0.18	50	3.6	2.91
D	-5	-5	-5	-5	-5	0.04	0.8	0.12
